# Structures of bimetallic Fe^III^–Fe^III^ and trimetallic Fe^III^–K–Fe^III^ complexes incorporating the deprotonated ligand *N*,*N*′-bis­(2-hy­droxy-3-methoxy­ben­zyl­idene)-1,2-phenyl­enedi­amine

**DOI:** 10.1107/S2056989026009734

**Published:** 2026-09-22

**Authors:** Mariama Thiam, Ngoné Diouf, James Orton, Cheikh Ndoye, Amadou Gueye, Ousmane Diouf, Ibrahima Elhadji Thiam, Mohamed Gaye, Simon Coles

**Affiliations:** a Département de Chimie, Faculté des Sciences et Techniques, Université Cheikh Anta Diop, Dakar, Senegal; b UK National Crystallography Service, School of Chemistry, Faculty of Engineering and Physical Sciences, University of Southampton, Southampton SO17 1BJ, United Kingdom; University of Aberdeen, United Kingdom

**Keywords:** iron, potassium, complex, crystal structure, square pyramidal

## Abstract

The title compounds μ-oxido-bis­[(2-{[2-(3-meth­oxy-2-oxido­benzyl­idene­amino)­phenyl­imino]­meth­yl}-6-meth­oxy­phenolato)iron(III)] penta­hydrate, [Fe_2_(C_22_H_18_N_2_O_4_]·5H_2_O or {[(Fe^III^(*L*)](μ_2_-O)[Fe^III^(*L*)]}·5H_2_O (**2**), and di­chlorido­bis­(μ-2-{[2-(3-meth­oxy-2-oxido­benzyl­idene­amino)­phenyl­imino]­meth­yl}-6-meth­oxy­phenolato)diiron(III)potassium(I) nitrate, [KFe_2_(C_22_H_18_N_2_O_4_)_2_Cl_2_]NO_3_ or {[(Fe^III^(*L*)(Cl)](K)[Fe^III^(*L*)(Cl)]}·NO_3_ (**3**), were formed from the reaction of the metallo-ligand [Fe^II^(*L*)]·(H_2_O) (H_2_*L* is 2-{[2-(2-hy­droxy-3-meth­oxy­benzyl­idene­amino)­phenyl­imino]­meth­yl}-6-meth­oxy­phenol, C_22_H_20_N_2_O_4_) with yttrium acetate and potassium nitrate, respectively. In complex **2**, the Fe^III^ ions, which presumably arise through aerial oxidation from Fe^II^, have a distorted square-pyramidal environment and are bridged by a μ_2_ oxo oxygen atom with an Fe—O—Fe angle of 137.97 (5)°. In complex (**3**) the environment around the Fe^III^ ions is a distorted square pyramid. All four phenoxo oxygen atoms link the Fe^III^ ions to a potassium atom (site symmetry 2), yielding two four-membered Fe/O/K/O rings. The K^+^ ion is linked also to four meth­oxy oxygen atoms.

## Abstract 2

The title compounds were formed from the reaction of the metallo-ligand [Fe^II^(*L*)]·(H_2_O) (H_2_*L* is 2-{[2-(2-hy­droxy-3-meth­oxy­benzyl­idene­amino)­phenyl­imino]­meth­yl}-6-meth­oxy­phenol, C_22_H_20_N_2_O_4_) with yttrium acetate and potassium nitrate.

## 1. Chemical context

Metalloligands are widely used precursors to prepare homopolynuclear and heteropolynuclear complexes. The resulting compounds, with properties such as mol­ecular magnetism (Braun *et al.*, 2024 ▸), biomedicine (Sun *et al.*, 2024 ▸), catalysis (Moree *et al.*, 2024 ▸) or luminescence (Shanmugavel *et al.*, 2024 ▸; Yazhini *et al.*, 2024 ▸) have been reported in the literature. The insertion of diamagnetic species such as alkali metal ions can give valuable information on the magneto-structural relationship of these compounds (Mason *et al.*, 2010 ▸). Access to these polynuclear compounds incorporating *ns* ions and 3*d* ions with different oxidation states, *e.g.*, sodium or potassium and iron(II, III) is a challenge from a synthetic point of view (Pinkert *et al.*, 2013 ▸). Chemists are increasingly inter­ested in the design and synthesis of heteropolynuclear complexes of *d*- and *s*-block metals (*e.g*., Thiam *et al.*, 2023 ▸). Indeed, the ligands used have two compartments of different sizes and the 3*d* element occupies the smaller cavity (Haba *et al.*, 2020*a* ▸,*b* ▸; Sarr *et al.*, 2018*a* ▸,*b* ▸). Various *s*-, *p*-, *d*- or *f*-type elements can occupy, in a second step, the larger cavity to form a dinuclear complex. The use of *s*-block elements in coordination chemistry has developed in recent years given their presence in biomolecular recognition (Kaczmarek *et al.*, 2018 ▸).
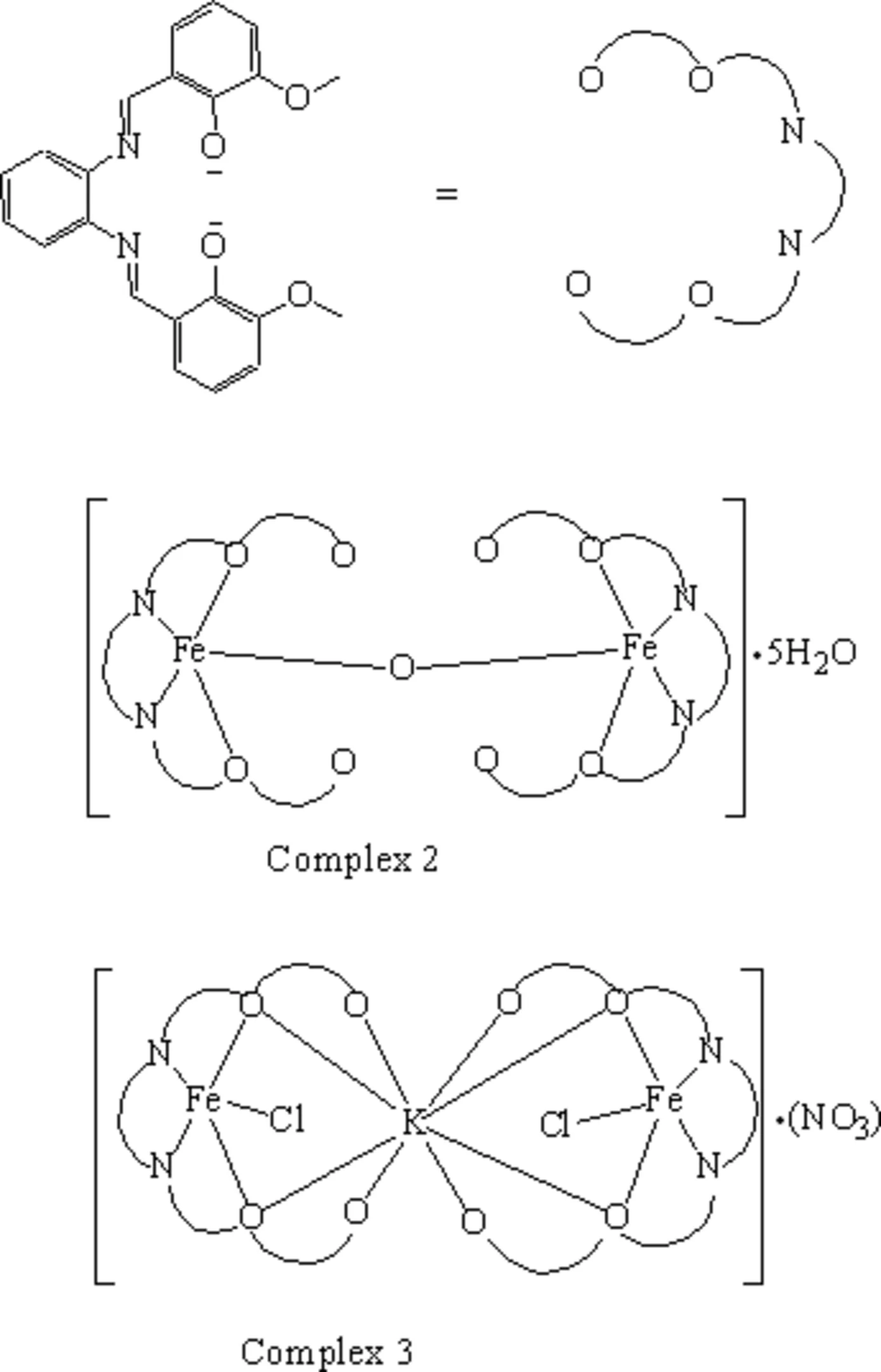


Our synthetic approach is to use an iron(II)-based metalloligand [Fe^II^(*L*)]·(H_2_O) synthesized from the reaction of the bi-compartmentalized Schiff ligand H_2_*L* (C_22_H_20_N_2_O_4_) derived from *o*-vanillin and 1,2-di­amino­benzene with FeCl_2_ under aerobic conditions (the composition [Fe^III^(*L*)Cl]·(H_2_O) is also possible, if the iron is oxidised by atmospheric oxygen during the synthesis). In this paper, we describe the synthesis, characterisation and structure of a homodinuclear complex {[(Fe(*L*)](μ_2_-O)[Fe(*L*)]}·5H_2_O (**2**) and a *d*/*s*/*d* heterotrinuclear complex formulated as {[(Fe(*L*)(Cl)](K)[Fe(*L*)(Cl)]}·NO_3_ (**3**).

## 2. Structural commentary

Complex **2** displays a mol­ecular structure (Fig. 1 ▸) constructed from two [Fe^III^(*L*)Cl] entities bridged by a μ_2_-O^2–^ ion, giving rise to a dinuclear neutral complex. The asymmetric unit contains two di-deprotonated tetra­dentate ligand mol­ecules, two penta­coordinated Fe^III^ ions, one O^2–^ ion acting in μ_2_- mode and five uncoordinated water mol­ecules. The Fe^III^ was presumably formed by aerial oxidation. The arrangement of the ligand donor atoms leads to the formation of an ‘inter­nal chamber’ defined by an N_2_O_2_ donor set and an outer chamber defined by O_2_O′_2_. The iron atoms are located in the smaller cavity (N_2_O_2_) and the larger cavity (O_2_O′_2_) is empty. Each iron atom is penta­coordinated by two azomethine nitro­gen atoms and two phenolate oxygen atoms and are bridged by one dianionic oxygen atom. The environment around the iron atom can be described using the Addison *et al.* (1984 ▸) τ parameter: when τ = 0, the geometry is a pyramid with a perfect square base; τ = 1 indicates a perfect (regular) trigonal bipyramidal geometry. The τ values of the Fe1 and the Fe2 atoms in **2** are, respectively, 0.002 and 0.208, which are indicative of a distorted square pyramidal around each iron atom. The distortion is greater around the Fe2 atom. The basal planes are defined by the atoms N1/N2/O2/O3 for Fe1 and N3/N4/O6/O7 for Fe2. The pyramids share one vertex occupied by the bridging oxygen atom O9 that is on the axial positions of the two square pyramids and the Fe1—O9—Fe2 bond angle is 137.97 (5) °. The *cis* angle values, for both Fe atoms, are in the range 76.28 (4)–93.50 (4)°, while the *trans* angles are in the range 140.16 (4)–152.65 (4)°. These values deviate substanti­ally from the ideal values of 90 and 180° expected for perfect square pyramidal geometry. Upon coordination, each ligand mol­ecule forms one five membered chelate ring and two six-membered rings. The atoms defining the five and the six membered rings are close to coplanar with r.m.s. deviations of 0.122 Å (Fe1/N1/C8/C13/N2), 0.086 Å (Fe1/O3/C20/C15/C14/N2), 0.112 Å (Fe1/N1/C7/C6/C1/O2) and 0.144 Å (Fe2/N3/C30/C35/N4), 0.120 Å (Fe2/O6/C23/C28/C29/N3) and 0.038 Å (Fe2/O7/C42/C37/C36/N4). Around Fe1 the mean plane of the five membered ring forms dihedral angles of 18.053 (3) and 16.825 (4)° with the mean planes of the six-membered rings. The six-membered rings form a dihedral angle of 27.896 (2)°. For Fe2 the mean plane of the five membered ring form dihedral angles of 19.527 (4) and 20.966 (3)° with the mean planes of the six-membered rings while the six-membered rings form a dihedral angle of 37.448 (2)°. The Fe1⋯Fe2 separation *via* the oxo bridge is 3.3350 (4) Å. The Fe—N distances (Table 1 ▸) are comparable to the values reported for similar complexes (*e.g*., Jana *et al.*, 2012 ▸). The Fe—O_p_ (p = phenoxo) distances are shorter the the distances Fe—O_b_ (b = bridge). These values are comparable to the values reported for similar complexes (Strauss *et al.*, 1987 ▸; Jana *et al.*, 2012 ▸).

The asymmetric unit of **3** contains a di-deprotonated *L*^2–^ ligand mol­ecule, an Fe^III^ cation, a K^+^ cation (site symmetry 2), an iron-bonded chloride anion and a free (uncoordinated) nitrate anion (Fig. 2 ▸). The complete complex is generated by crystallographic twofold symmetry. In the structure of the trinuclear complex, each of the Fe atoms is linked to the K atom by two phenoxo oxygen atoms. The Fe^III^ ion, which occupies the smaller inner pocket of the ligand, is coordinated in a square pyramidal geometry with a basal plane formed by two azomethine nitro­gen atoms and two phenoxo oxygen atoms. The apical position is occupied by a chloride ion. The *cissoid* angles around the iron atom, which are in the range 77.94 (3)–89.74 (3)°, deviate significantly from the ideal values of 90°. The diagonal basal angles which are, respectively, N1—Fe1—O3 = 146.34 (3)° and N2—Fe1—O1 = 150.97 (3)° deviate from the ideal values of 180°. These values are comparable to those reported for similar complexes (Haba *et al.*, 2020*a* ▸,*b* ▸). The Fe1 atom is significantly displaced from the plane defined by the N_2_O_2_ site, the out-of-plane distance being 0.418 (3) Å. In the basal planes of the square pyramid, the Fe—O_p_ distances are the smallest (Table 2 ▸) while the Fe—N_i_ (i = imino) bond distances are the longest. The Fe1—Cl1 distance is comparable to the values reported for similar complexes (Safo *et al.*, 2010 ▸; Awasabisah *et al.*, 2015 ▸; Senge, 2005 ▸). The potassium ion (site symmetry 2), which occupies the larger open site O_2_O′_2_ of the ligand, is ligated to two phenoxo oxygen atoms and two meth­oxy oxygen atoms per ligand mol­ecule. Thus, by symmetry, the potassium cation is situated in a distorted KO_8_ polyhedron site made up by four μ_2_-bridging phenolate oxygen atoms and four meth­oxy oxygen atoms from two ligand mol­ecules. The K—O_p_ bond distances are the shortest distances (Fig. 2 ▸, Table 2 ▸) while the K1—O_m_ (m = meth­oxy) are the longest. These distances are shorter than those reported for a complex in which iron and potassium ions are bridged by a cyano group (Huang *et al.*, 2019 ▸). The C1–C6 phenyl group in **3** is almost coplanar with the C16–C21 phenyl ring and is slightly twisted from the second phenyl ring with meth­oxy group with dihedral angle values of 0.386 (6)° and 5.515 (5)°, respectively. The phenyl rings bearing meth­oxy groups form a dihedral angle of 5.552 (5)°. The potassium ion is sandwiched between the iron atoms with Fe1⋯K1 = 3.7246 (2) Å and Fe1⋯K1⋯Fe1^i^ = 178.44 (9)° [symmetry code: (i) 1 − *x*, *y*, 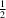 − *z*], this distance being slightly longer than those found in a similar heteronuclear Fe/K complex (Purdy & Butcher, 2014 ▸). The O atoms of the nitrate counter-ion are disordered over two orientations rotated by ∼120°.

## 3. Supra­molecular features

In the extended structure of **2**, numerous O—H⋯O hydrogen bonds are observed (Fig. 3 ▸, Table 3 ▸). These are made possible by the presence of the free (uncoordinated) water mol­ecules, which link the complex entities. Projection down the [101] direction reveals the resulting three-dimensional supra­molecular framework (Fig. 4 ▸).

Examination of the supra­molecular network of complex **3** reveals six weak hydrogen bonds (Table 4 ▸). The nitrate and chloride ions act as bridges between neighbouring complex mol­ecules *via* C—H⋯O and C—H⋯Cl hydrogen bonds (Fig. 5 ▸). These inter­actions ensure the cohesion of a three-dimensional network which, when projected down [101], appears as *pseudo* layers (Fig. 6 ▸).

## 4. Database survey

A search of the Cambridge Structural Database (CSD, version 5.46; February 2025 update; Groom *et al.*, 2016 ▸), carried out on 1 April 2026, for complexes displaying a coordination motif similar to that of **2** and **3** returned eleven hits with the following CSD refcodes: AGASOR (Nabei *et al.*, 2008 ▸), AGASOR01 (Nabei *et al.*, 2008 ▸), BEHJEF (Jana *et al.*, 2012 ▸), BEHJIJ (Jana *et al.*, 2012 ▸), KESLAZ (Elemo *et al.*, 2022 ▸), WEHGAT (Bhattacharya *et al.*, 2012 ▸), WEHGEX (Bhattacharya *et al.*, 2012 ▸), XATYAW (Haba *et al.*, 2020*a* ▸,*b* ▸), XOFRES (Raj *et al.*, 2019 ▸), XOFRIW (Raj *et al.*, 2019 ▸), and XOFROC (Raj *et al.*, 2019 ▸). These compounds exhibit a range of nuclearities, including mononuclear species (*e.g*., BEHJEF, KESLAZ, and XOFRIW) and dinuclear species (*e.g*., BEHJIJ and XOFRES), the latter frequently featuring μ-oxo bridges between Fe^III^ centres. In addition, polymeric chain structures are observed for AGASOR and AGASOR01, whereas heterometallic systems incorporating vanadium are reported for WEHGAT and WEHGEX. Despite this structural diversity, all of these compounds share a common tetra­dentate *N*,*N*,*O*,*O* coordination motif.

## 5. Synthesis and crystallization

[Fe^III^(*L*)Cl]·(H_2_O) **1**. In a round-bottomed flask, the ligand (H_2_*L*) (10 mmol, 0.374 g) was dissolved in 5 ml of aceto­nitrile. A solution of FeCl_2_·4H_2_O (10 mmol, 0.199 g) in 5 ml of methanol was added. After two h under reflux, the brown precipitate was recovered by filtration, washed with ether (2 × 10 ml) and dried in air.

Yield: 80%. Elemental analysis found (calculated) (%): C, 58.92 (58.95); H, 4.48 (4.50); N, 6.22 (2.25). IR (ν, cm^−1^): 3343 (ν_O—H_), 1627 (ν_C=N_), 1236 (ν_C–OPh_), 1201, (ν_C–OMe_), 851 (δH_2_O). Conductance (S cm^2^ mol^−1^): 7.00.

{[(Fe^III^(*L*)](μ_2_-O)[Fe^III^(*L*)]}·5H_2_O **2**. To a solution of (**1**) (0.25 mmol, 0.1234 g) in 5 ml of *N*,*N*-di­methyl­formamide (DMF) was added a solution of Y(CH_3_CO_2_)_3_ (0.25 mmol, 0.0665 g) in 5 ml of ethanol. The mixture was refluxed for two h. On cooling, a black precipitate appeared, which was recovered by filtration. The filtrate was left for slow evaporation. On standing for two weeks, black crystals of (**2**) suitable for X-ray diffraction were isolated. Yield: 69%. Elemental analysis found (calculated) (%): C, 54.68 (54.64); H, 4.80 (4.76); N, 5.80 (5.78); Cl, 6.87 (6.84). IR (ν, cm^−1^): 1601 (ν_C=N_), 1239 (ν_C–OPh_), 1196 (ν_C–OMe_). Conductance (S cm^2^ mol^−1^): 6.80.

{[(Fe^III^(*L*)(Cl)](K)[Fe^III^(*L*)(Cl)]}·NO_3_**3**. To a solution of (**1**) (0.25 mmol, 0.1234 g) in 5 ml of DMF was added a solution of KNO_3_ (0.25 mmol, 0.0225 g) in 5 ml of ethanol. The mixture was refluxed for two h. On cooling, a black precipitate was recovered by filtration. The filtrate was left for slow evaporation. On standing for two weeks, black crystals of (**3**) suitable for X-ray diffraction were isolated. Yield: 58%. Elemental analysis found (calculated) (%): C, 51.18 (51.15); H, 3.51 (3.49); N, 6.78 (6.75); Cl, 6.87 (6.84). IR (ν, cm^−1^): 1600 (ν_C=N_), 1201 (ν_C-OPh_), 1105 (ν_C-OMe_). Conductance (S cm^2^ mol^−1^): 61.0.

## 6. Refinement

Crystal data, data collection and structure refinement details are summarized in Table 5 ▸. The C-bound H atoms were positioned geometrically (0.95–0.98 Å) and refined as riding with *U*_iso_(H) = 1.2*U*_eq_(C). The other H atoms were located in difference maps and refined as riding with *U*_iso_(H) = 1.2*U*_eq_(N) or 1.5*U*_eq_(O).

## Supplementary Material

Crystal structure: contains datablock(s) 3, 2. DOI: 10.1107/S2056989026009734/hb8251sup1.cif

Structure factors: contains datablock(s) 3. DOI: 10.1107/S2056989026009734/hb82513sup2.hkl

Structure factors: contains datablock(s) 2. DOI: 10.1107/S2056989026009734/hb82512sup3.hkl

CCDC references: 2588878, 2588877

Additional supporting information:  crystallographic information; 3D view; checkCIF report

## Figures and Tables

**Figure 1:**
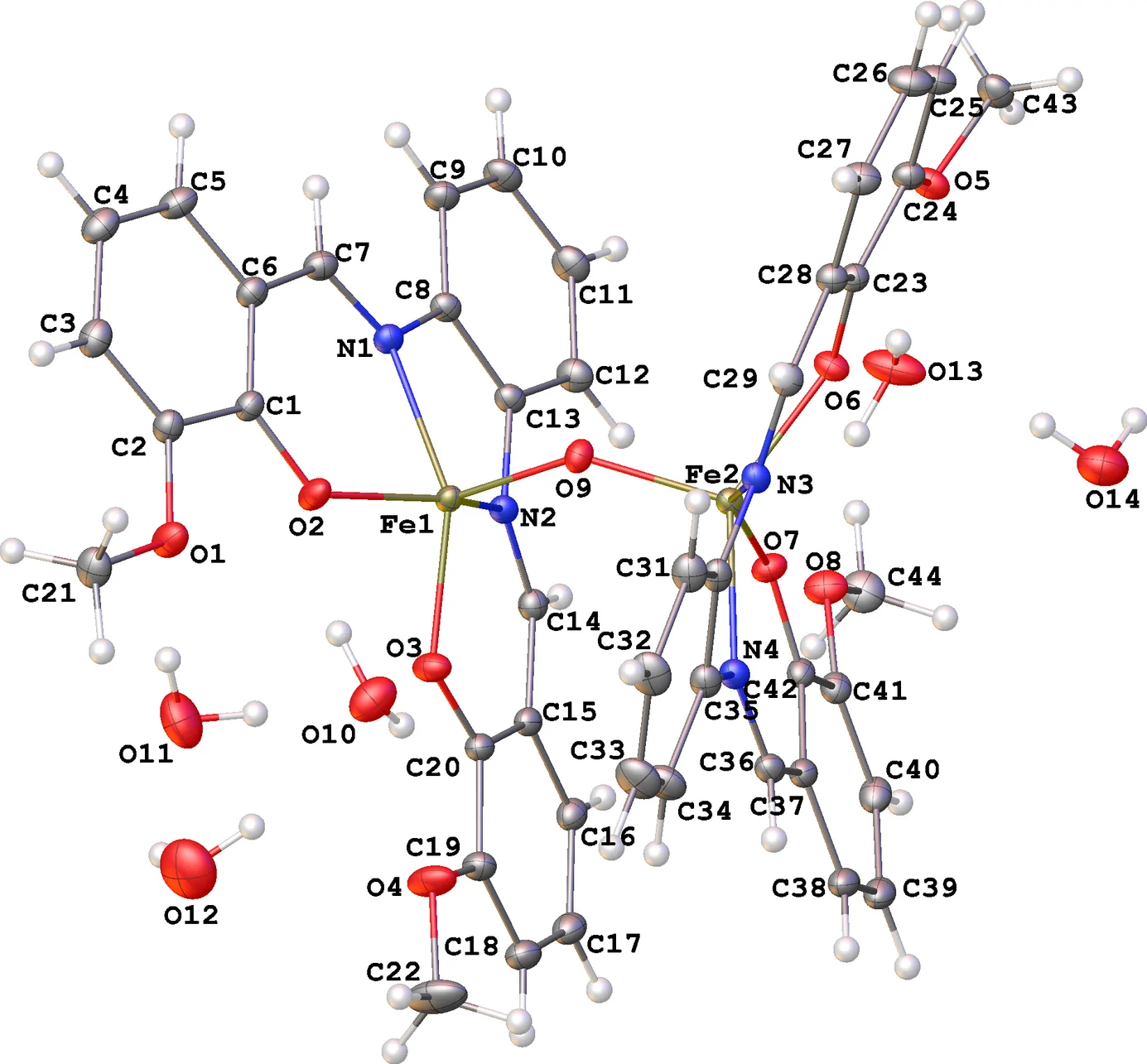
The mol­ecular structure of **2** with 50% probability ellipsoids.

**Figure 2:**
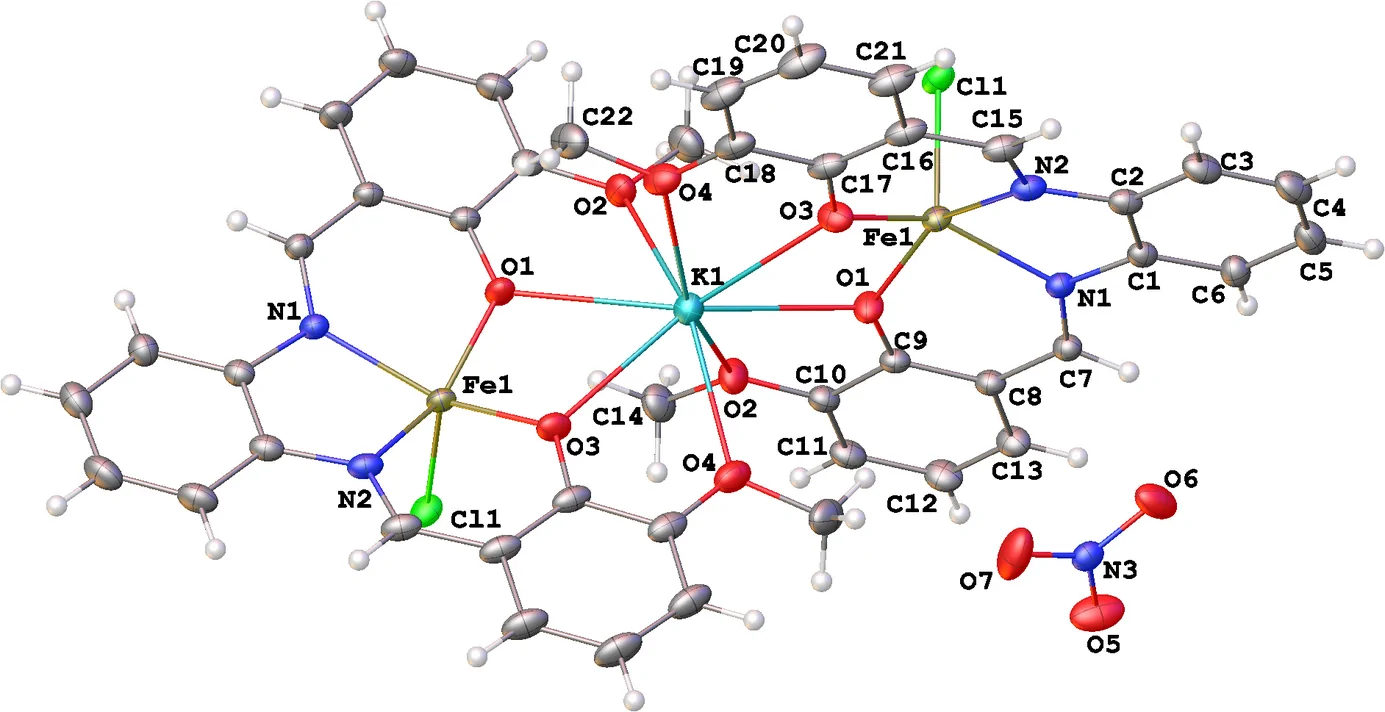
The mol­ecular structure of **3** with 50% probability ellipsoids.

**Figure 3:**
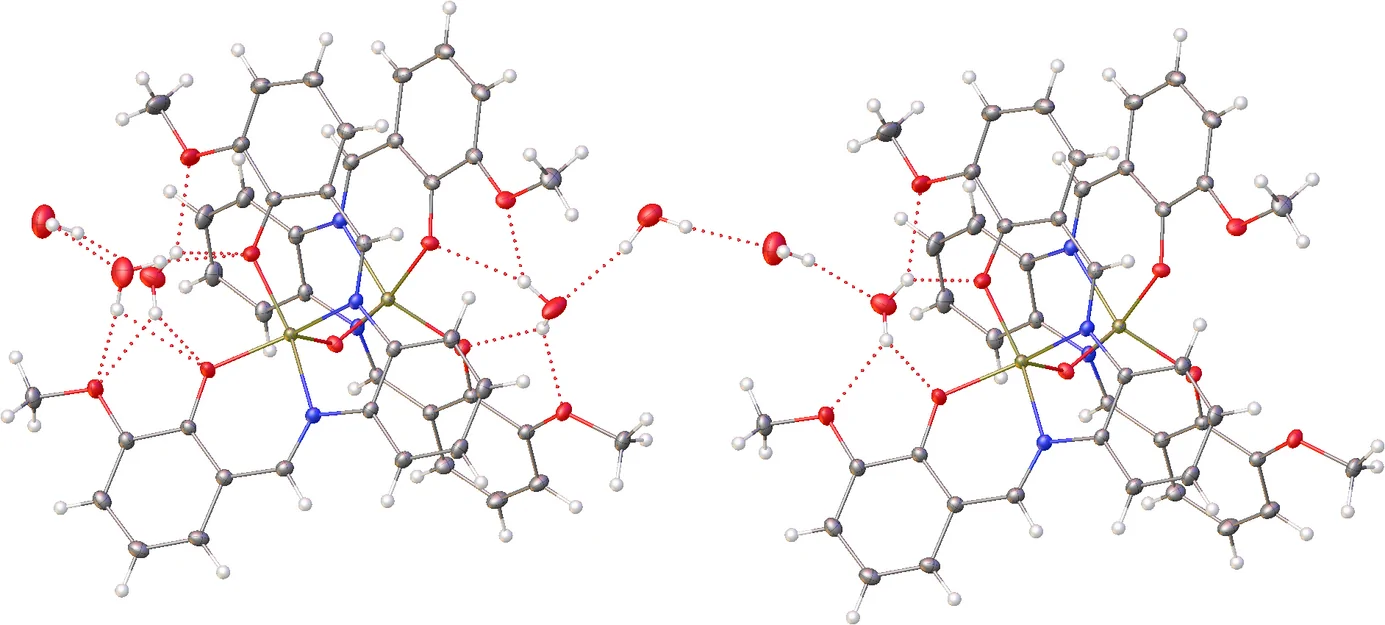
A fragment of the extended structure of **2** showing hydrogen bonds as dashed lines.

**Figure 4:**
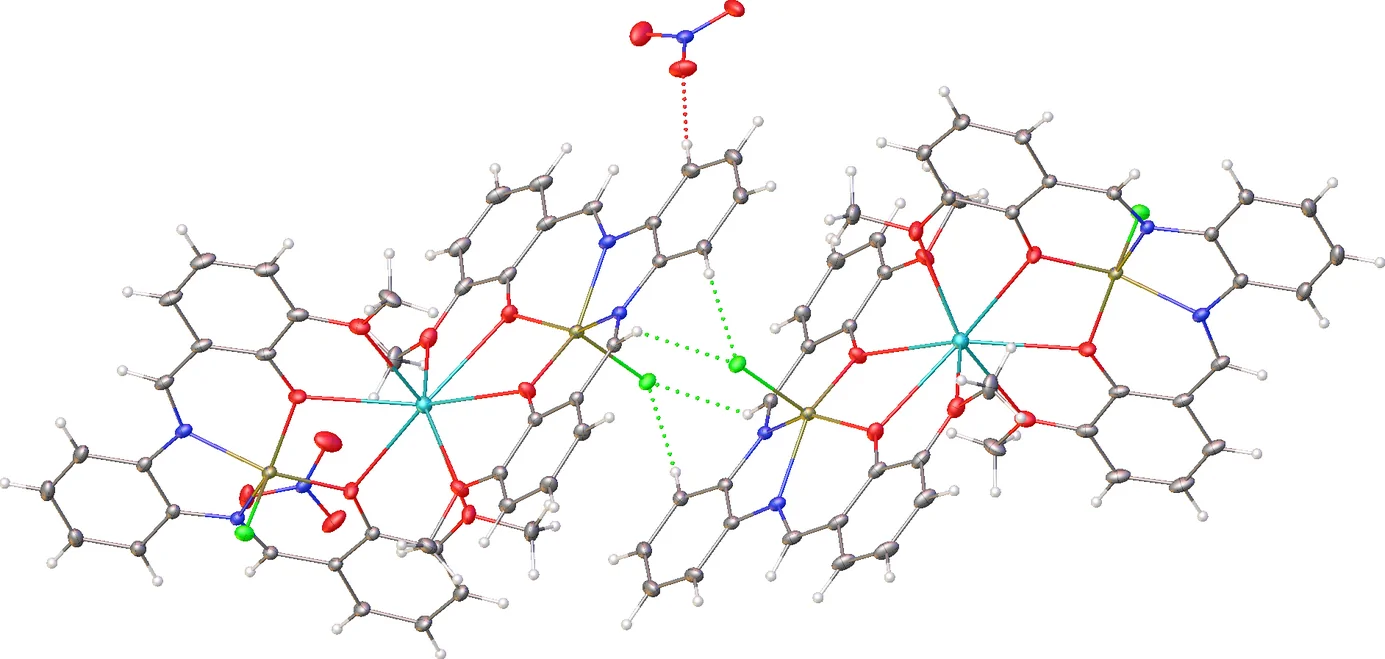
The packing of **2** viewed down [101]. H atoms not involved in hydrogen bonds are omitted for clarity.

**Figure 5:**
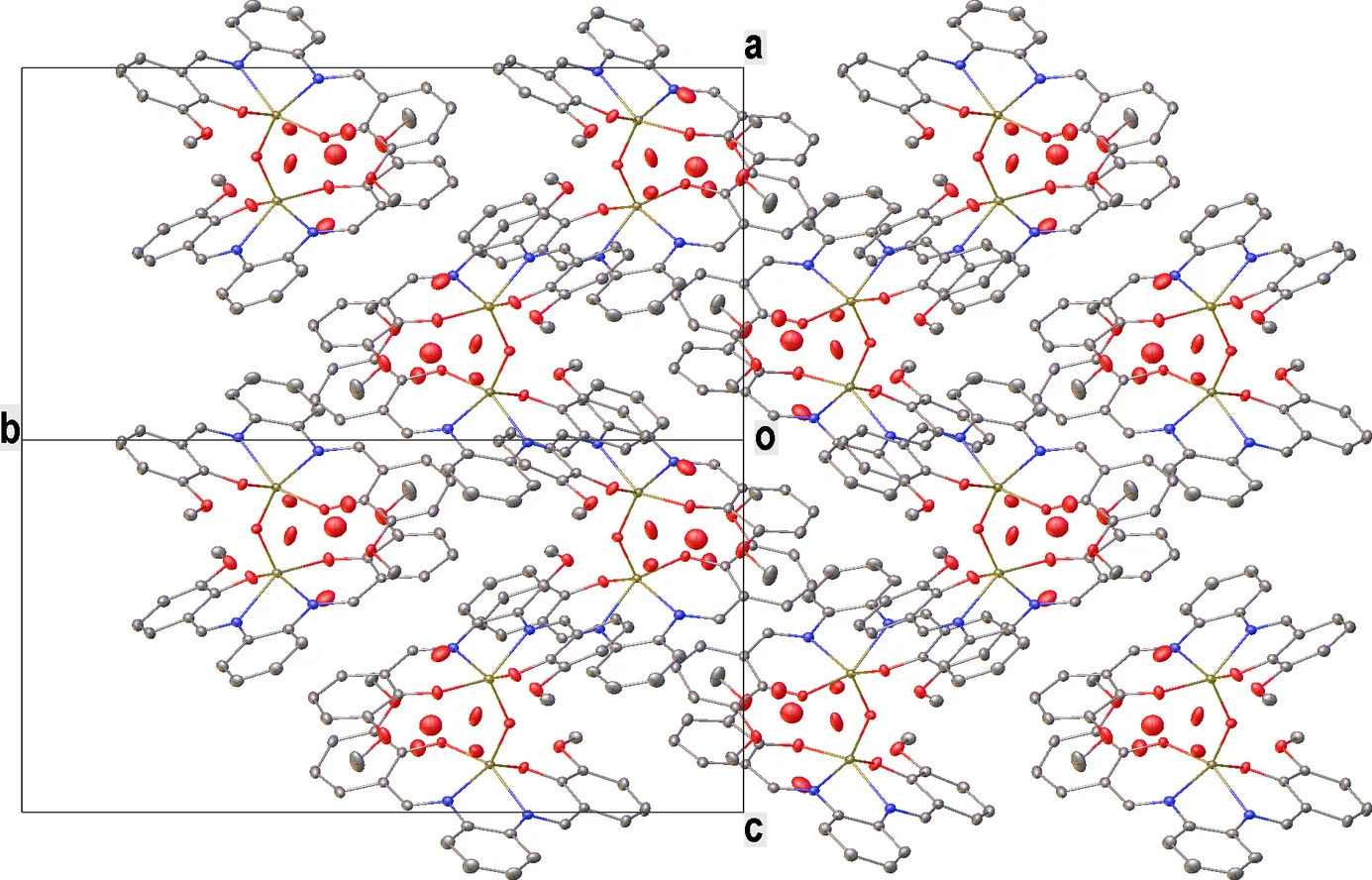
A fragment of the extended structure of **3** showing hydrogen bonds as dashed lines.

**Figure 6:**
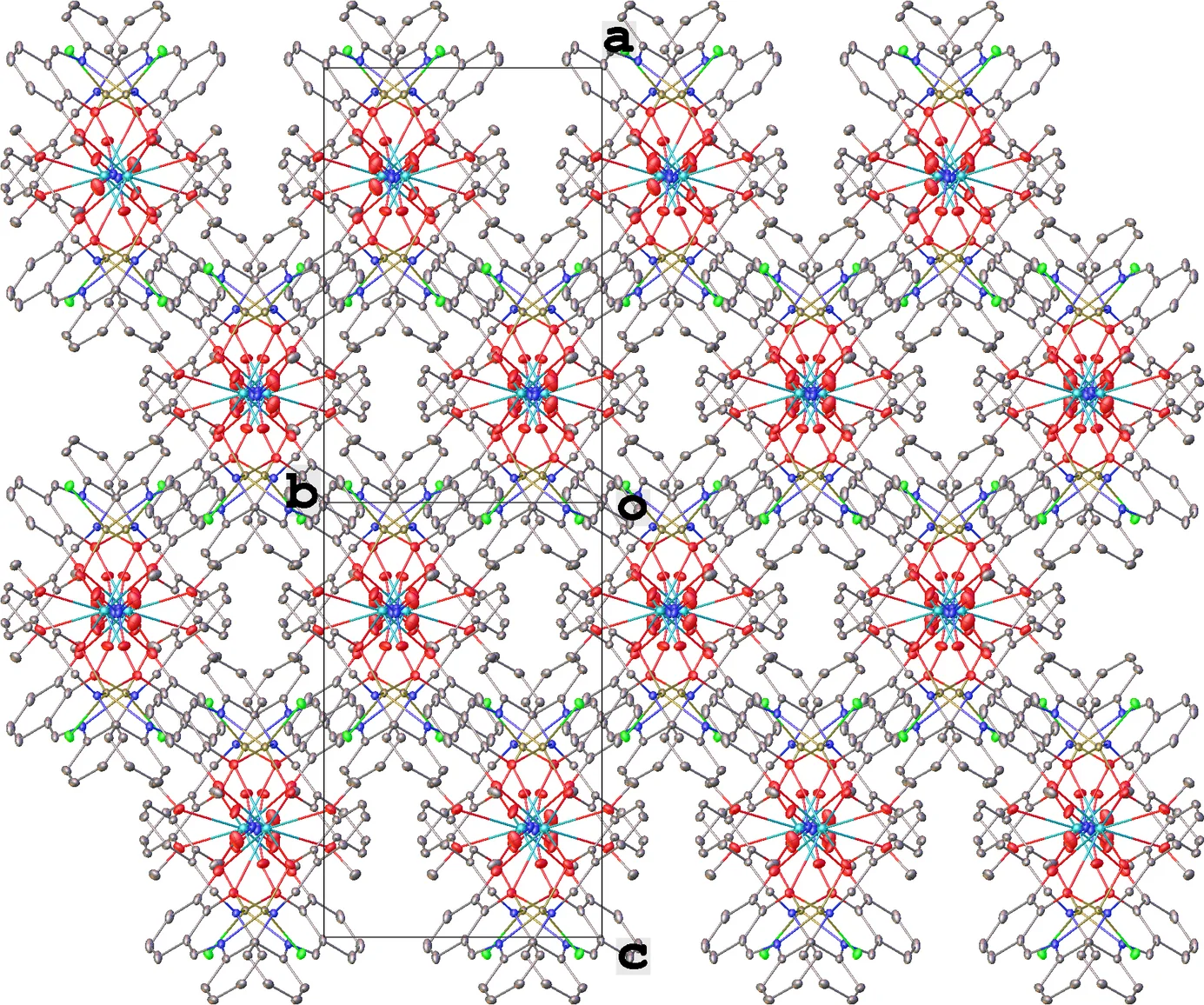
The packing of **3** viewed down [101]. H atoms not involved in hydrogen bonds are omitted for clarity.

**Table 1:** Selected bond lengths (Å) for **2**[Chem scheme1]

Fe1—N1	2.1035 (10)	Fe2—N3	2.1232 (10)
Fe1—N2	2.0893 (10)	Fe2—N4	2.1036 (10)
Fe1—O2	1.9373 (9)	Fe2—O6	1.9374 (8)
Fe1—O3	1.9262 (9)	Fe2—O7	1.9246 (9)
Fe1—O9	1.7835 (9)	Fe2—O9	1.7893 (8)

**Table 2:** Selected bond lengths (Å) for **3**[Chem scheme1]

Fe1—Cl1	2.2285 (3)	K1—O1	2.7476 (7)
Fe1—N1	2.0705 (8)	K1—O2	2.7900 (8)
Fe1—N2	2.0848 (8)	K1—O3	2.7497 (8)
Fe1—O1	1.9010 (7)	K1—O4	2.7890 (8)
Fe1—O3	1.8980 (7)		

**Table 3:** Hydrogen-bond geometry (Å, °) for **2**[Chem scheme1]

*D*—H⋯*A*	*D*—H	H⋯*A*	*D*⋯*A*	*D*—H⋯*A*
C7—H7⋯O10^i^	0.95	2.60	3.5233 (16)	165
C36—H36⋯O14^ii^	0.95	2.45	3.3930 (17)	172
C44—H44*B*⋯O11^iii^	0.98	2.58	3.4643 (19)	151
O10—H10*A*⋯O1	0.87	2.21	2.9342 (15)	140
O10—H10*A*⋯O2	0.87	2.28	3.0393 (14)	146
O10—H10*B*⋯O3	0.87	2.42	3.1432 (14)	141
O10—H10*B*⋯O4	0.87	2.28	3.0579 (15)	148
O11—H11*A*⋯O3	0.87	2.11	2.9635 (15)	169
O11—H11*B*⋯O1	0.87	2.48	3.2974 (16)	157
O11—H11*B*⋯O2	0.87	2.52	3.0582 (15)	121
O12—H12*A*⋯O10	0.87	2.00	2.8520 (19)	167
O12—H12*B*⋯O11	0.87	2.23	2.9457 (19)	139
O13—H13*A*⋯O5	0.87	2.11	2.8255 (14)	139
O13—H13*A*⋯O6	0.87	2.27	3.0237 (13)	145
O13—H13*B*⋯O7	0.87	2.30	3.0580 (14)	146
O13—H13*B*⋯O8	0.87	2.08	2.8108 (14)	141
O14—H14*A*⋯O12^iv^	0.87	1.99	2.8457 (17)	167
O14—H14*B*⋯O13	0.87	2.01	2.8683 (18)	167

Symmetry codes: (i) 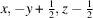; (ii) 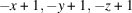; (iii) 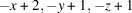; (iv) 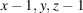.

**Table 4:** Hydrogen-bond geometry (Å, °) for **3**[Chem scheme1]

*D*—H⋯*A*	*D*—H	H⋯*A*	*D*⋯*A*	*D*—H⋯*A*
C3—H3⋯O5^i^	0.95	2.28	3.221 (2)	170
C6—H6⋯Cl1^ii^	0.95	2.81	3.7493 (10)	170
C7—H7⋯Cl1^ii^	0.95	2.88	3.6840 (9)	143
C15—H15⋯O6^iii^	0.95	2.38	3.2611 (19)	155
C19—H19⋯Cl1^iv^	0.95	2.70	3.5639 (11)	151
C22—H22*A*⋯O7^v^	0.98	2.41	2.908 (2)	111

Symmetry codes: (i) 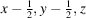; (ii) 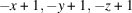; (iii) 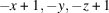; (iv) 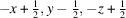; (v) 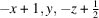.

**Table 5:** Experimental details

	**2**	**3**
Crystal data		
Chemical formula	[Fe_2_(C_22_H_18_N_2_O_4_)_2_O]·5H_2_O	[KFe_2_(C_22_H_18_N_2_O_4_)_2_Cl_2_]NO_3_
*M*_r_	966.55	1032.48
Crystal system, space group	Monoclinic, *P*2_1_/*c*	Monoclinic, *C*2/*c*
Temperature (K)	100	100
*a*, *b*, *c* (Å)	13.6001 (4), 22.9342 (5), 14.8502 (5)	17.5443 (8), 10.0392 (5), 24.4944 (13)
β (°)	113.199 (4)	103.438 (5)
*V* (Å^3^)	4257.4 (2)	4196.1 (4)
*Z*	4	4
Radiation type	Mo *K*α	Mo *K*α
μ (mm^−1^)	0.76	0.99
Crystal size (mm)	0.14 × 0.13 × 0.02	0.22 × 0.12 × 0.06

Data collection		
Diffractometer	Rigaku FRE+	Rigaku FRE+
Absorption correction	Analytical (*CrysAlis PRO*; Rigaku OD, 2024 ▸)	Analytical (*CrysAlis PRO*; Rigaku OD, 2024 ▸)
*T*_min_, *T*_max_	0.915, 0.985	0.888, 0.952
No. of measured, independent and observed [*I* > 2σ(*I*)] reflections	99080, 20596, 15233	59339, 10162, 8768
*R*_int_	0.049	0.028
(sin θ/λ)_max_ (Å^−1^)	0.833	0.833

Refinement		
*R*[*F*^2^ > 2σ(*F*^2^)], *wR*(*F*^2^), *S*	0.044, 0.119, 1.04	0.031, 0.087, 1.04
No. of reflections	20596	10162
No. of parameters	596	314
H-atom treatment	H-atom parameters constrained	H-atom parameters constrained
Δρ_max_, Δρ_min_ (e Å^−3^)	0.70, −0.66	0.57, −0.32

Computer programs: *CrysAlis PRO* (Rigaku OD, 2024 ▸), *SHELXT2018/2* (Sheldrick, 2015*a* ▸), *SHELXL2018/3* (Sheldrick, 2015*b* ▸) and *OLEX2* (Dolomanov *et al.*, 2009 ▸).

## supplementary crystallographic information

### Dichloridobis(µ-2-{[2-(3-methoxy-2-oxidobenzylideneamino)phenylimino]methyl}-6-methoxyphenolato)diiron(III)potassium(I) nitrate (3) Crystal data

**Table:**

[KFe_2_(C_22_H_18_N_2_O_4_)_2_Cl_2_]NO_3_	*F*(000) = 2112
*M**_r_* = 1032.48	*D*_x_ = 1.634 Mg m^−^^3^
Monoclinic, *C*2/*c*	Mo *K*α radiation, λ = 0.71075 Å
*a* = 17.5443 (8) Å	Cell parameters from 6255 reflections
*b* = 10.0392 (5) Å	θ = 2.4–38.0°
*c* = 24.4944 (13) Å	µ = 0.99 mm^−^^1^
β = 103.438 (5)°	*T* = 100 K
*V* = 4196.1 (4) Å^3^	(cut) irregular block, dark-red
*Z* = 4	0.22 × 0.12 × 0.06 mm

### Dichloridobis(µ-2-{[2-(3-methoxy-2-oxidobenzylideneamino)phenylimino]methyl}-6-methoxyphenolato)diiron(III)potassium(I) nitrate (3) Data collection

**Table:**

Rigaku FRE+ diffractometer	8768 reflections with *I* > 2σ(*I*)
Detector resolution: 10 pixels mm^-1^	*R*_int_ = 0.028
profile data from ω–scans	θ_max_ = 36.3°, θ_min_ = 1.7°
Absorption correction: analytical (CrysAlisPro; Rigaku OD, 2024)	*h* = −29→29
*T*_min_ = 0.888, *T*_max_ = 0.952	*k* = −16→16
59339 measured reflections	*l* = −40→40
10162 independent reflections	

### Dichloridobis(µ-2-{[2-(3-methoxy-2-oxidobenzylideneamino)phenylimino]methyl}-6-methoxyphenolato)diiron(III)potassium(I) nitrate (3) Refinement

**Table:**

Refinement on *F*^2^	Primary atom site location: dual
Least-squares matrix: full	Hydrogen site location: inferred from neighbouring sites
*R*[*F*^2^ > 2σ(*F*^2^)] = 0.031	H-atom parameters constrained
*wR*(*F*^2^) = 0.087	*w* = 1/[σ^2^(*F*_o_^2^) + (0.050*P*)^2^ + 1.8043*P*] where *P* = (*F*_o_^2^ + 2*F*_c_^2^)/3
*S* = 1.04	(Δ/σ)_max_ = 0.001
10162 reflections	Δρ_max_ = 0.57 e Å^−^^3^
314 parameters	Δρ_min_ = −0.32 e Å^−^^3^
0 restraints	

### Dichloridobis(µ-2-{[2-(3-methoxy-2-oxidobenzylideneamino)phenylimino]methyl}-6-methoxyphenolato)diiron(III)potassium(I) nitrate (3) Special details

**Table:**

Geometry. All esds (except the esd in the dihedral angle between two l.s. planes) are estimated using the full covariance matrix. The cell esds are taken into account individually in the estimation of esds in distances, angles and torsion angles; correlations between esds in cell parameters are only used when they are defined by crystal symmetry. An approximate (isotropic) treatment of cell esds is used for estimating esds involving l.s. planes.

### Dichloridobis(µ-2-{[2-(3-methoxy-2-oxidobenzylideneamino)phenylimino]methyl}-6-methoxyphenolato)diiron(III)potassium(I) nitrate (3) Fractional atomic coordinates and isotropic or equivalent isotropic displacement parameters (Å^2^)

**Table:**

	*x*	*y*	*z*	*U*_iso_*/*U*_eq_	Occ. (<1)
C1	0.50193 (5)	0.24785 (9)	0.51306 (4)	0.01710 (13)	
C2	0.44651 (5)	0.14641 (9)	0.49579 (4)	0.01895 (14)	
C3	0.41983 (6)	0.07184 (10)	0.53609 (5)	0.02564 (18)	
H3	0.382175	0.003222	0.524806	0.031*	
C4	0.44890 (7)	0.09925 (11)	0.59237 (5)	0.0285 (2)	
H4	0.431973	0.047444	0.619790	0.034*	
C5	0.50242 (7)	0.20127 (10)	0.60941 (4)	0.02561 (18)	
H5	0.520826	0.220049	0.648257	0.031*	
C6	0.52919 (6)	0.27592 (9)	0.57017 (4)	0.02096 (15)	
H6	0.565848	0.345765	0.581977	0.025*	
C7	0.58541 (5)	0.39691 (9)	0.48030 (4)	0.01716 (13)	
H7	0.613315	0.403463	0.518398	0.021*	
C8	0.61348 (5)	0.47516 (8)	0.43993 (4)	0.01674 (13)	
C9	0.58326 (5)	0.46279 (8)	0.38173 (4)	0.01670 (13)	
C10	0.61495 (5)	0.54446 (9)	0.34517 (4)	0.01898 (14)	
C11	0.67262 (6)	0.63660 (9)	0.36649 (4)	0.02232 (16)	
H11	0.692316	0.692528	0.341650	0.027*	
C12	0.70231 (6)	0.64806 (10)	0.42471 (5)	0.02456 (17)	
H12	0.742020	0.711618	0.439103	0.029*	
C13	0.67411 (5)	0.56770 (9)	0.46086 (4)	0.02173 (16)	
H13	0.695403	0.574201	0.500138	0.026*	
C14	0.60618 (7)	0.61086 (12)	0.25021 (5)	0.0291 (2)	
H14A	0.662778	0.603632	0.253535	0.044*	
H14B	0.593072	0.702596	0.258218	0.044*	
H14C	0.578244	0.586882	0.212015	0.044*	
C15	0.37662 (5)	0.03054 (9)	0.41536 (5)	0.02230 (16)	
H15	0.360109	−0.028962	0.440487	0.027*	
C16	0.34971 (5)	0.00534 (9)	0.35632 (5)	0.02312 (17)	
C17	0.37616 (5)	0.07742 (9)	0.31471 (4)	0.02150 (16)	
C18	0.34533 (5)	0.04398 (9)	0.25756 (5)	0.02402 (17)	
C19	0.29085 (6)	−0.05724 (10)	0.24331 (5)	0.0287 (2)	
H19	0.270229	−0.078458	0.204915	0.034*	
C20	0.26583 (6)	−0.12884 (10)	0.28516 (5)	0.0317 (2)	
H20	0.228562	−0.198452	0.274915	0.038*	
C21	0.29477 (6)	−0.09903 (10)	0.34067 (5)	0.0285 (2)	
H21	0.277917	−0.148565	0.368758	0.034*	
C22	0.32925 (7)	0.11073 (13)	0.16200 (5)	0.0328 (2)	
H22A	0.350967	0.173913	0.139136	0.049*	
H22B	0.274081	0.132198	0.159616	0.049*	
H22C	0.333272	0.020085	0.148094	0.049*	
Cl1	0.34951 (2)	0.41749 (2)	0.38730 (2)	0.02194 (5)	
Fe1	0.45267 (2)	0.28570 (2)	0.39054 (2)	0.01585 (3)	
K1	0.500000	0.29194 (3)	0.250000	0.02250 (5)	
N1	0.52506 (4)	0.31785 (7)	0.46914 (3)	0.01594 (11)	
N2	0.42196 (4)	0.12888 (7)	0.43695 (3)	0.01895 (13)	
N3	0.7457 (13)	0.2407 (17)	0.5001 (11)	0.0190 (12)	0.5
O5	0.80190 (10)	0.32155 (18)	0.51234 (10)	0.0389 (4)	0.5
O6	0.70583 (11)	0.21995 (18)	0.53603 (8)	0.0349 (4)	0.5
O7	0.73006 (15)	0.1847 (2)	0.45496 (8)	0.0451 (5)	0.5
O1	0.52741 (4)	0.37741 (7)	0.35940 (3)	0.02128 (12)	
O2	0.58350 (4)	0.52265 (8)	0.28938 (3)	0.02515 (14)	
O3	0.42868 (4)	0.17378 (7)	0.32638 (3)	0.02267 (13)	
O4	0.37251 (4)	0.11956 (8)	0.21957 (3)	0.02677 (14)	

### Dichloridobis(µ-2-{[2-(3-methoxy-2-oxidobenzylideneamino)phenylimino]methyl}-6-methoxyphenolato)diiron(III)potassium(I) nitrate (3) Atomic displacement parameters (Å^2^)

**Table:**

	*U*^11^	*U*^22^	*U*^33^	*U*^12^	*U*^13^	*U*^23^
C1	0.0157 (3)	0.0154 (3)	0.0206 (3)	0.0017 (2)	0.0051 (3)	0.0021 (3)
C2	0.0158 (3)	0.0153 (3)	0.0265 (4)	0.0013 (3)	0.0065 (3)	0.0023 (3)
C3	0.0224 (4)	0.0214 (4)	0.0360 (5)	−0.0009 (3)	0.0125 (4)	0.0058 (3)
C4	0.0283 (5)	0.0284 (5)	0.0335 (5)	0.0027 (4)	0.0165 (4)	0.0083 (4)
C5	0.0290 (4)	0.0264 (4)	0.0244 (4)	0.0060 (3)	0.0121 (4)	0.0041 (3)
C6	0.0232 (4)	0.0200 (4)	0.0203 (4)	0.0017 (3)	0.0063 (3)	0.0007 (3)
C7	0.0157 (3)	0.0171 (3)	0.0185 (3)	−0.0012 (2)	0.0034 (3)	−0.0017 (3)
C8	0.0141 (3)	0.0155 (3)	0.0208 (3)	−0.0018 (2)	0.0044 (3)	−0.0018 (3)
C9	0.0136 (3)	0.0157 (3)	0.0210 (3)	−0.0010 (2)	0.0045 (3)	−0.0005 (3)
C10	0.0164 (3)	0.0186 (3)	0.0229 (4)	−0.0005 (3)	0.0067 (3)	0.0014 (3)
C11	0.0192 (3)	0.0194 (4)	0.0304 (4)	−0.0028 (3)	0.0100 (3)	0.0009 (3)
C12	0.0213 (4)	0.0210 (4)	0.0325 (5)	−0.0076 (3)	0.0086 (3)	−0.0037 (3)
C13	0.0195 (3)	0.0206 (4)	0.0249 (4)	−0.0065 (3)	0.0049 (3)	−0.0048 (3)
C14	0.0331 (5)	0.0290 (5)	0.0279 (5)	−0.0012 (4)	0.0123 (4)	0.0082 (4)
C15	0.0159 (3)	0.0138 (3)	0.0353 (5)	−0.0008 (3)	0.0021 (3)	0.0011 (3)
C16	0.0159 (3)	0.0134 (3)	0.0365 (5)	−0.0004 (3)	−0.0012 (3)	−0.0023 (3)
C17	0.0145 (3)	0.0152 (3)	0.0320 (4)	0.0007 (3)	−0.0002 (3)	−0.0063 (3)
C18	0.0175 (3)	0.0180 (4)	0.0333 (5)	0.0028 (3)	−0.0007 (3)	−0.0073 (3)
C19	0.0222 (4)	0.0194 (4)	0.0382 (5)	0.0006 (3)	−0.0061 (4)	−0.0083 (4)
C20	0.0237 (4)	0.0179 (4)	0.0463 (6)	−0.0037 (3)	−0.0064 (4)	−0.0043 (4)
C21	0.0201 (4)	0.0159 (4)	0.0445 (6)	−0.0030 (3)	−0.0027 (4)	−0.0017 (4)
C22	0.0277 (5)	0.0418 (6)	0.0285 (5)	−0.0015 (4)	0.0054 (4)	−0.0173 (4)
Cl1	0.02003 (9)	0.02151 (9)	0.02147 (9)	0.00524 (7)	−0.00090 (7)	−0.00103 (7)
Fe1	0.01339 (5)	0.01431 (5)	0.01887 (6)	−0.00220 (4)	0.00173 (4)	−0.00074 (4)
K1	0.02232 (12)	0.02485 (13)	0.01974 (11)	0.000	0.00370 (9)	0.000
N1	0.0141 (3)	0.0148 (3)	0.0185 (3)	−0.0004 (2)	0.0027 (2)	0.0008 (2)
N2	0.0143 (3)	0.0141 (3)	0.0275 (4)	−0.0006 (2)	0.0029 (2)	0.0001 (2)
N3	0.019 (4)	0.015 (4)	0.0219 (5)	0.000 (2)	0.002 (2)	0.003 (3)
O5	0.0257 (8)	0.0244 (7)	0.0633 (13)	−0.0068 (7)	0.0041 (8)	0.0092 (8)
O6	0.0318 (8)	0.0357 (9)	0.0428 (10)	0.0080 (6)	0.0203 (7)	0.0171 (7)
O7	0.0592 (13)	0.0449 (10)	0.0240 (8)	0.0127 (10)	−0.0048 (8)	−0.0069 (7)
O1	0.0194 (3)	0.0242 (3)	0.0193 (3)	−0.0085 (2)	0.0027 (2)	−0.0004 (2)
O2	0.0263 (3)	0.0291 (3)	0.0204 (3)	−0.0065 (3)	0.0060 (2)	0.0042 (3)
O3	0.0193 (3)	0.0214 (3)	0.0271 (3)	−0.0056 (2)	0.0049 (2)	−0.0073 (2)
O4	0.0210 (3)	0.0279 (3)	0.0292 (4)	−0.0006 (3)	0.0012 (3)	−0.0088 (3)

### Dichloridobis(µ-2-{[2-(3-methoxy-2-oxidobenzylideneamino)phenylimino]methyl}-6-methoxyphenolato)diiron(III)potassium(I) nitrate (3) Geometric parameters (Å, º)

**Table:**

C1—C2	1.4038 (12)	C16—C17	1.4132 (15)
C1—C6	1.3979 (13)	C16—C21	1.4149 (13)
C1—N1	1.4208 (11)	C17—C18	1.4188 (14)
C2—C3	1.4032 (13)	C17—O3	1.3206 (11)
C2—N2	1.4159 (13)	C18—C19	1.3831 (14)
C3—H3	0.9500	C18—O4	1.3694 (14)
C3—C4	1.3818 (17)	C19—H19	0.9500
C4—H4	0.9500	C19—C20	1.4034 (18)
C4—C5	1.3866 (16)	C20—H20	0.9500
C5—H5	0.9500	C20—C21	1.3694 (17)
C5—C6	1.3840 (14)	C21—H21	0.9500
C6—H6	0.9500	C22—H22A	0.9800
C7—H7	0.9500	C22—H22B	0.9800
C7—C8	1.4365 (12)	C22—H22C	0.9800
C7—N1	1.3005 (11)	C22—O4	1.4404 (14)
C8—C9	1.4063 (12)	Fe1—Cl1	2.2285 (3)
C8—C13	1.4156 (12)	Fe1—K1	3.7246 (2)
C9—C10	1.4194 (12)	Fe1—N1	2.0705 (8)
C9—O1	1.3209 (10)	Fe1—N2	2.0848 (8)
C10—C11	1.3807 (13)	Fe1—O1	1.9010 (7)
C10—O2	1.3674 (12)	Fe1—O3	1.8980 (7)
C11—H11	0.9500	K1—O1^i^	2.7476 (7)
C11—C12	1.4048 (15)	K1—O1	2.7476 (7)
C12—H12	0.9500	K1—O2^i^	2.7900 (8)
C12—C13	1.3722 (14)	K1—O2	2.7900 (8)
C13—H13	0.9500	K1—O3	2.7497 (8)
C14—H14A	0.9800	K1—O3^i^	2.7497 (8)
C14—H14B	0.9800	K1—O4	2.7890 (8)
C14—H14C	0.9800	K1—O4^i^	2.7889 (8)
C14—O2	1.4284 (12)	N3—O5	1.258 (15)
C15—H15	0.9500	N3—O6	1.26 (2)
C15—C16	1.4356 (15)	N3—O7	1.21 (2)
C15—N2	1.3006 (12)		
			
C2—C1—N1	115.48 (8)	H22B—C22—H22C	109.5
C6—C1—C2	119.99 (8)	O4—C22—H22A	109.5
C6—C1—N1	124.51 (8)	O4—C22—H22B	109.5
C1—C2—N2	115.00 (8)	O4—C22—H22C	109.5
C3—C2—C1	119.73 (9)	Cl1—Fe1—K1	108.107 (8)
C3—C2—N2	125.27 (9)	N1—Fe1—Cl1	104.96 (2)
C2—C3—H3	120.3	N1—Fe1—K1	129.53 (2)
C4—C3—C2	119.31 (9)	N1—Fe1—N2	77.94 (3)
C4—C3—H3	120.3	N2—Fe1—Cl1	99.03 (2)
C3—C4—H4	119.5	N2—Fe1—K1	131.16 (2)
C3—C4—C5	120.96 (9)	O1—Fe1—Cl1	109.17 (2)
C5—C4—H4	119.5	O1—Fe1—K1	45.44 (2)
C4—C5—H5	119.8	O1—Fe1—N1	88.07 (3)
C6—C5—C4	120.42 (10)	O1—Fe1—N2	150.97 (3)
C6—C5—H5	119.8	O3—Fe1—Cl1	107.46 (2)
C1—C6—H6	120.2	O3—Fe1—K1	45.48 (2)
C5—C6—C1	119.56 (9)	O3—Fe1—N1	146.34 (3)
C5—C6—H6	120.2	O3—Fe1—N2	88.21 (3)
C8—C7—H7	117.2	O3—Fe1—O1	89.74 (3)
N1—C7—H7	117.2	O1^i^—K1—O1	143.60 (3)
N1—C7—C8	125.57 (8)	O1^i^—K1—O2	92.10 (2)
C9—C8—C7	122.73 (7)	O1—K1—O2^i^	92.11 (2)
C9—C8—C13	119.97 (8)	O1^i^—K1—O2^i^	56.27 (2)
C13—C8—C7	117.30 (8)	O1—K1—O2	56.27 (2)
C8—C9—C10	118.55 (8)	O1^i^—K1—O3^i^	58.36 (2)
O1—C9—C8	123.07 (8)	O1^i^—K1—O3	142.57 (2)
O1—C9—C10	118.37 (8)	O1—K1—O3^i^	142.57 (2)
C11—C10—C9	120.56 (8)	O1—K1—O3	58.36 (2)
O2—C10—C9	114.32 (8)	O1^i^—K1—O4	89.16 (2)
O2—C10—C11	125.12 (8)	O1—K1—O4^i^	89.17 (2)
C10—C11—H11	119.8	O1—K1—O4	113.71 (2)
C10—C11—C12	120.34 (9)	O1^i^—K1—O4^i^	113.71 (2)
C12—C11—H11	119.8	O2—K1—O2^i^	67.77 (3)
C11—C12—H12	119.9	O3—K1—O2^i^	107.94 (2)
C13—C12—C11	120.17 (9)	O3^i^—K1—O2^i^	114.10 (2)
C13—C12—H12	119.9	O3^i^—K1—O2	107.93 (2)
C8—C13—H13	119.8	O3—K1—O2	114.10 (2)
C12—C13—C8	120.37 (9)	O3^i^—K1—O3	128.89 (3)
C12—C13—H13	119.8	O3—K1—O4	56.68 (2)
H14A—C14—H14B	109.5	O3^i^—K1—O4	90.80 (2)
H14A—C14—H14C	109.5	O3^i^—K1—O4^i^	56.67 (2)
H14B—C14—H14C	109.5	O3—K1—O4^i^	90.80 (2)
O2—C14—H14A	109.5	O4—K1—O2	158.68 (2)
O2—C14—H14B	109.5	O4^i^—K1—O2	95.66 (2)
O2—C14—H14C	109.5	O4^i^—K1—O2^i^	158.68 (2)
C16—C15—H15	117.6	O4—K1—O2^i^	95.66 (2)
N2—C15—H15	117.6	O4^i^—K1—O4	103.29 (3)
N2—C15—C16	124.78 (9)	C1—N1—Fe1	113.93 (5)
C17—C16—C15	123.36 (8)	C7—N1—C1	120.30 (8)
C17—C16—C21	120.07 (10)	C7—N1—Fe1	125.74 (6)
C21—C16—C15	116.56 (10)	C2—N2—Fe1	113.90 (6)
C16—C17—C18	118.46 (8)	C15—N2—C2	121.40 (8)
O3—C17—C16	123.27 (9)	C15—N2—Fe1	124.24 (7)
O3—C17—C18	118.27 (9)	O5—N3—O6	118.1 (19)
C19—C18—C17	120.37 (10)	O7—N3—O5	121.1 (18)
O4—C18—C17	115.23 (8)	O7—N3—O6	120.9 (12)
O4—C18—C19	124.40 (10)	C9—O1—Fe1	131.50 (6)
C18—C19—H19	119.8	C9—O1—K1	123.46 (5)
C18—C19—C20	120.47 (10)	Fe1—O1—K1	105.03 (3)
C20—C19—H19	119.8	C10—O2—C14	117.47 (8)
C19—C20—H20	119.8	C10—O2—K1	122.41 (5)
C21—C20—C19	120.44 (9)	C14—O2—K1	119.23 (6)
C21—C20—H20	119.8	C17—O3—Fe1	128.48 (7)
C16—C21—H21	119.9	C17—O3—K1	125.01 (6)
C20—C21—C16	120.19 (11)	Fe1—O3—K1	105.04 (3)
C20—C21—H21	119.9	C18—O4—C22	116.34 (9)
H22A—C22—H22B	109.5	C18—O4—K1	123.13 (6)
H22A—C22—H22C	109.5	C22—O4—K1	120.48 (7)
			
C1—C2—C3—C4	−0.26 (14)	C16—C15—N2—Fe1	8.87 (13)
C1—C2—N2—C15	173.39 (8)	C16—C17—C18—C19	−0.20 (13)
C1—C2—N2—Fe1	−14.17 (9)	C16—C17—C18—O4	178.84 (8)
C2—C1—C6—C5	1.35 (13)	C16—C17—O3—Fe1	−25.98 (13)
C2—C1—N1—C7	−167.93 (8)	C16—C17—O3—K1	169.98 (6)
C2—C1—N1—Fe1	14.15 (9)	C17—C16—C21—C20	−1.32 (15)
C2—C3—C4—C5	1.61 (16)	C17—C18—C19—C20	−0.49 (15)
C3—C2—N2—C15	−6.72 (14)	C17—C18—O4—C22	−165.44 (8)
C3—C2—N2—Fe1	165.73 (7)	C17—C18—O4—K1	11.77 (11)
C3—C4—C5—C6	−1.49 (16)	C18—C17—O3—Fe1	154.58 (7)
C4—C5—C6—C1	−0.01 (15)	C18—C17—O3—K1	−9.47 (11)
C6—C1—C2—C3	−1.22 (13)	C18—C19—C20—C21	0.28 (16)
C6—C1—C2—N2	178.68 (8)	C19—C18—O4—C22	13.56 (14)
C6—C1—N1—C7	13.54 (13)	C19—C18—O4—K1	−169.23 (7)
C6—C1—N1—Fe1	−164.39 (7)	C19—C20—C21—C16	0.62 (16)
C7—C8—C9—C10	−179.90 (8)	C21—C16—C17—C18	1.10 (13)
C7—C8—C9—O1	−0.81 (13)	C21—C16—C17—O3	−178.35 (9)
C7—C8—C13—C12	−178.34 (9)	Cl1—Fe1—O3—C17	−68.02 (8)
C8—C7—N1—C1	−178.51 (8)	Cl1—Fe1—O3—K1	98.49 (2)
C8—C7—N1—Fe1	−0.85 (13)	K1—Fe1—O3—C17	−166.52 (10)
C8—C9—C10—C11	−1.82 (13)	N1—C1—C2—C3	−179.82 (8)
C8—C9—C10—O2	178.20 (8)	N1—C1—C2—N2	0.08 (11)
C8—C9—O1—Fe1	19.15 (13)	N1—C1—C6—C5	179.82 (8)
C8—C9—O1—K1	−159.48 (6)	N1—C7—C8—C9	−7.64 (14)
C9—C8—C13—C12	1.69 (14)	N1—C7—C8—C13	172.39 (9)
C9—C10—C11—C12	1.83 (14)	N1—Fe1—O3—C17	95.79 (9)
C9—C10—O2—C14	173.29 (8)	N1—Fe1—O3—K1	−97.69 (5)
C9—C10—O2—K1	−17.59 (10)	N2—C2—C3—C4	179.85 (9)
C10—C9—O1—Fe1	−161.76 (7)	N2—C15—C16—C17	6.75 (15)
C10—C9—O1—K1	19.61 (11)	N2—C15—C16—C21	−174.19 (9)
C10—C11—C12—C13	−0.05 (15)	N2—Fe1—O3—C17	30.88 (8)
C11—C10—O2—C14	−6.70 (14)	N2—Fe1—O3—K1	−162.60 (3)
C11—C10—O2—K1	162.42 (7)	O1—C9—C10—C11	179.05 (8)
C11—C12—C13—C8	−1.71 (15)	O1—C9—C10—O2	−0.94 (12)
C13—C8—C9—C10	0.07 (12)	O1—Fe1—O3—C17	−178.07 (8)
C13—C8—C9—O1	179.16 (8)	O1—Fe1—O3—K1	−11.56 (3)
C15—C16—C17—C18	−179.87 (8)	O2—C10—C11—C12	−178.18 (9)
C15—C16—C17—O3	0.69 (14)	O3—C17—C18—C19	179.27 (9)
C15—C16—C21—C20	179.58 (9)	O3—C17—C18—O4	−1.69 (12)
C16—C15—N2—C2	−179.48 (8)	O4—C18—C19—C20	−179.44 (9)

Symmetry code: (i) −*x*+1, *y*, −*z*+1/2.

### Dichloridobis(µ-2-{[2-(3-methoxy-2-oxidobenzylideneamino)phenylimino]methyl}-6-methoxyphenolato)diiron(III)potassium(I) nitrate (3) Hydrogen-bond geometry (Å, º)

**Table:**

*D*—H···*A*	*D*—H	H···*A*	*D*···*A*	*D*—H···*A*
C3—H3···O5^ii^	0.95	2.28	3.221 (2)	170
C6—H6···Cl1^iii^	0.95	2.81	3.7493 (10)	170
C7—H7···Cl1^iii^	0.95	2.88	3.6840 (9)	143
C15—H15···O6^iv^	0.95	2.38	3.2611 (19)	155
C19—H19···Cl1^v^	0.95	2.70	3.5639 (11)	151
C22—H22*A*···O7^i^	0.98	2.41	2.908 (2)	111

Symmetry codes: (i) −*x*+1, *y*, −*z*+1/2; (ii) *x*−1/2, *y*−1/2, *z*; (iii) −*x*+1, −*y*+1, −*z*+1; (iv) −*x*+1, −*y*, −*z*+1; (v) −*x*+1/2, *y*−1/2, −*z*+1/2.

### µ-Oxido-bis[(2-{[2-(3-methoxy-2-oxidobenzylideneamino)phenylimino]methyl}-6-methoxyphenolato)iron(III)] pentahydrate, (2) Crystal data

**Table:**

[Fe_2_(C_22_H_18_N_2_O_4_)_2_O]·5H_2_O	*F*(000) = 2008
*M**_r_* = 966.55	*D*_x_ = 1.508 Mg m^−^^3^
Monoclinic, *P*2_1_/*c*	Mo *K*α radiation, λ = 0.71075 Å
*a* = 13.6001 (4) Å	Cell parameters from 11083 reflections
*b* = 22.9342 (5) Å	θ = 1.7–37.9°
*c* = 14.8502 (5) Å	µ = 0.76 mm^−^^1^
β = 113.199 (4)°	*T* = 100 K
*V* = 4257.4 (2) Å^3^	(cut) lath, dark-red
*Z* = 4	0.14 × 0.13 × 0.02 mm

### µ-Oxido-bis[(2-{[2-(3-methoxy-2-oxidobenzylideneamino)phenylimino]methyl}-6-methoxyphenolato)iron(III)] pentahydrate, (2) Data collection

**Table:**

Rigaku FRE+ diffractometer	15233 reflections with *I* > 2σ(*I*)
Detector resolution: 10 pixels mm^-1^	*R*_int_ = 0.049
profile data from ω–scans	θ_max_ = 36.3°, θ_min_ = 1.6°
Absorption correction: analytical (CrysAlisPro; Rigaku OD, 2024)	*h* = −22→22
*T*_min_ = 0.915, *T*_max_ = 0.985	*k* = −38→38
99080 measured reflections	*l* = −24→24
20596 independent reflections	

### µ-Oxido-bis[(2-{[2-(3-methoxy-2-oxidobenzylideneamino)phenylimino]methyl}-6-methoxyphenolato)iron(III)] pentahydrate, (2) Refinement

**Table:**

Refinement on *F*^2^	Primary atom site location: dual
Least-squares matrix: full	Hydrogen site location: mixed
*R*[*F*^2^ > 2σ(*F*^2^)] = 0.044	H-atom parameters constrained
*wR*(*F*^2^) = 0.119	*w* = 1/[σ^2^(*F*_o_^2^) + (0.0651*P*)^2^ + 0.4018*P*] where *P* = (*F*_o_^2^ + 2*F*_c_^2^)/3
*S* = 1.04	(Δ/σ)_max_ = 0.001
20596 reflections	Δρ_max_ = 0.70 e Å^−^^3^
596 parameters	Δρ_min_ = −0.66 e Å^−^^3^
0 restraints	

### µ-Oxido-bis[(2-{[2-(3-methoxy-2-oxidobenzylideneamino)phenylimino]methyl}-6-methoxyphenolato)iron(III)] pentahydrate, (2) Special details

**Table:**

Geometry. All esds (except the esd in the dihedral angle between two l.s. planes) are estimated using the full covariance matrix. The cell esds are taken into account individually in the estimation of esds in distances, angles and torsion angles; correlations between esds in cell parameters are only used when they are defined by crystal symmetry. An approximate (isotropic) treatment of cell esds is used for estimating esds involving l.s. planes.

### µ-Oxido-bis[(2-{[2-(3-methoxy-2-oxidobenzylideneamino)phenylimino]methyl}-6-methoxyphenolato)iron(III)] pentahydrate, (2) Fractional atomic coordinates and isotropic or equivalent isotropic displacement parameters (Å^2^)

**Table:**

	*x*	*y*	*z*	*U*_iso_*/*U*_eq_	
C1	1.08876 (9)	0.26717 (5)	0.67740 (8)	0.01280 (19)	
C2	1.16248 (10)	0.24855 (5)	0.77116 (9)	0.0143 (2)	
C3	1.21656 (10)	0.19627 (6)	0.78313 (9)	0.0168 (2)	
H3	1.265479	0.184681	0.846347	0.020*	
C4	1.19921 (11)	0.16026 (6)	0.70187 (10)	0.0181 (2)	
H4	1.236234	0.124227	0.710186	0.022*	
C5	1.12871 (11)	0.17703 (6)	0.61019 (9)	0.0172 (2)	
H5	1.116761	0.152104	0.555782	0.021*	
C6	1.07372 (10)	0.23083 (5)	0.59571 (9)	0.01344 (19)	
C7	1.01093 (10)	0.24828 (5)	0.49648 (9)	0.0143 (2)	
H7	1.006415	0.221706	0.445933	0.017*	
C8	0.90690 (9)	0.31284 (5)	0.37006 (8)	0.01228 (19)	
C9	0.89805 (10)	0.27744 (6)	0.29048 (9)	0.0158 (2)	
H9	0.929948	0.239845	0.301535	0.019*	
C10	0.84288 (11)	0.29712 (6)	0.19561 (9)	0.0177 (2)	
H10	0.838676	0.273414	0.141756	0.021*	
C11	0.79343 (11)	0.35170 (6)	0.17892 (9)	0.0180 (2)	
H11	0.753453	0.364358	0.113722	0.022*	
C12	0.80232 (10)	0.38753 (6)	0.25697 (9)	0.0165 (2)	
H12	0.768362	0.424589	0.245216	0.020*	
C13	0.86130 (9)	0.36908 (5)	0.35292 (8)	0.01261 (19)	
C14	0.86585 (9)	0.45908 (5)	0.43142 (8)	0.01299 (19)	
H14	0.842477	0.476080	0.368075	0.016*	
C15	0.88340 (9)	0.49758 (5)	0.51263 (9)	0.01227 (19)	
C16	0.85687 (10)	0.55718 (5)	0.49079 (9)	0.0149 (2)	
H16	0.828358	0.569966	0.424586	0.018*	
C17	0.87220 (10)	0.59651 (5)	0.56485 (10)	0.0171 (2)	
H17	0.853918	0.636313	0.549479	0.020*	
C18	0.91454 (10)	0.57844 (6)	0.66279 (10)	0.0179 (2)	
H18	0.924599	0.605938	0.713507	0.021*	
C19	0.94174 (10)	0.52068 (5)	0.68585 (9)	0.0159 (2)	
C20	0.92776 (9)	0.47886 (5)	0.61106 (9)	0.01308 (19)	
C21	1.24369 (11)	0.27202 (6)	0.94121 (9)	0.0186 (2)	
H21A	1.315565	0.264844	0.943232	0.028*	
H21B	1.217041	0.236589	0.960896	0.028*	
H21C	1.246519	0.303823	0.986232	0.028*	
C22	0.99273 (16)	0.53626 (7)	0.85697 (11)	0.0332 (4)	
H22A	1.024290	0.515325	0.919384	0.050*	
H22B	0.921502	0.550476	0.847949	0.050*	
H22C	1.038435	0.569379	0.857384	0.050*	
C23	0.52840 (9)	0.25172 (5)	0.44578 (8)	0.01176 (18)	
C24	0.46778 (9)	0.22290 (5)	0.35609 (8)	0.01268 (19)	
C25	0.43012 (11)	0.16688 (6)	0.35462 (9)	0.0172 (2)	
H25	0.390311	0.148472	0.293762	0.021*	
C26	0.45041 (12)	0.13691 (6)	0.44277 (10)	0.0194 (2)	
H26	0.425375	0.098106	0.441344	0.023*	
C27	0.50633 (11)	0.16367 (6)	0.53081 (9)	0.0169 (2)	
H27	0.518748	0.143494	0.590172	0.020*	
C28	0.54589 (9)	0.22136 (5)	0.53402 (8)	0.01287 (19)	
C29	0.59648 (9)	0.24760 (5)	0.62881 (8)	0.01302 (19)	
H29	0.599779	0.225325	0.683861	0.016*	
C30	0.68372 (9)	0.32289 (5)	0.74144 (8)	0.01303 (19)	
C31	0.70575 (10)	0.29133 (6)	0.82799 (9)	0.0170 (2)	
H31	0.688947	0.250992	0.825223	0.020*	
C32	0.75210 (11)	0.31921 (7)	0.91752 (9)	0.0213 (3)	
H32	0.766979	0.297903	0.976298	0.026*	
C33	0.77717 (12)	0.37847 (7)	0.92208 (9)	0.0236 (3)	
H33	0.809004	0.397159	0.983975	0.028*	
C34	0.75600 (11)	0.41032 (6)	0.83700 (9)	0.0197 (2)	
H34	0.773706	0.450562	0.840548	0.024*	
C35	0.70840 (10)	0.38273 (5)	0.74597 (8)	0.0142 (2)	
C36	0.66511 (9)	0.46648 (5)	0.64641 (9)	0.0137 (2)	
H36	0.669278	0.486802	0.703553	0.016*	
C37	0.63824 (9)	0.49964 (5)	0.55805 (9)	0.01299 (19)	
C38	0.61599 (10)	0.55999 (5)	0.56164 (10)	0.0161 (2)	
H38	0.620872	0.576832	0.621696	0.019*	
C39	0.58763 (10)	0.59411 (5)	0.47960 (10)	0.0173 (2)	
H39	0.572358	0.634290	0.482858	0.021*	
C40	0.58109 (10)	0.56966 (5)	0.39037 (10)	0.0165 (2)	
H40	0.561737	0.593468	0.333634	0.020*	
C41	0.60270 (9)	0.51128 (5)	0.38511 (9)	0.0138 (2)	
C42	0.62963 (9)	0.47407 (5)	0.46857 (9)	0.01229 (19)	
C43	0.38309 (10)	0.23312 (6)	0.18210 (9)	0.0171 (2)	
H43A	0.372500	0.262585	0.131329	0.026*	
H43B	0.313892	0.222924	0.183858	0.026*	
H43C	0.415559	0.198218	0.167304	0.026*	
C44	0.57068 (12)	0.51586 (6)	0.21474 (10)	0.0216 (3)	
H44A	0.567734	0.489983	0.161224	0.032*	
H44B	0.623386	0.546720	0.223027	0.032*	
H44C	0.500134	0.533374	0.199164	0.032*	
Fe1	0.92344 (2)	0.35310 (2)	0.56529 (2)	0.01038 (4)	
Fe2	0.66903 (2)	0.35162 (2)	0.54108 (2)	0.00972 (4)	
N1	0.95992 (8)	0.29733 (4)	0.47026 (7)	0.01231 (17)	
N2	0.87934 (8)	0.40288 (4)	0.43754 (7)	0.01174 (17)	
N3	0.63825 (8)	0.29959 (4)	0.64536 (7)	0.01168 (16)	
N4	0.68429 (8)	0.41050 (4)	0.65444 (7)	0.01255 (17)	
O1	1.17380 (8)	0.28770 (4)	0.84418 (6)	0.01919 (18)	
O2	1.04159 (7)	0.31752 (4)	0.67200 (6)	0.01700 (17)	
O3	0.95949 (8)	0.42490 (4)	0.63837 (6)	0.01651 (17)	
O4	0.98394 (9)	0.49786 (4)	0.77871 (7)	0.0236 (2)	
O5	0.45184 (7)	0.25582 (4)	0.27481 (6)	0.01544 (16)	
O6	0.56115 (7)	0.30521 (4)	0.44185 (6)	0.01348 (15)	
O7	0.64462 (7)	0.41871 (4)	0.45718 (6)	0.01361 (15)	
O8	0.60097 (8)	0.48307 (4)	0.30340 (7)	0.01774 (17)	
O9	0.79937 (7)	0.32477 (4)	0.56055 (6)	0.01312 (15)	
O10	1.01072 (10)	0.37077 (5)	0.84647 (8)	0.0260 (2)	
H10A	1.033811	0.344794	0.816773	0.039*	
H10B	0.989409	0.399583	0.805018	0.039*	
O11	1.19078 (9)	0.42012 (6)	0.76496 (9)	0.0321 (3)	
H11A	1.124104	0.426667	0.727741	0.048*	
H11B	1.194509	0.382485	0.773752	0.048*	
O12	1.19790 (12)	0.43386 (7)	0.96466 (9)	0.0438 (3)	
H12A	1.135376	0.418501	0.932145	0.066*	
H12B	1.221847	0.442892	0.920136	0.066*	
O13	0.51035 (11)	0.37150 (5)	0.25280 (8)	0.0295 (3)	
H13A	0.502313	0.343602	0.288940	0.044*	
H13B	0.552296	0.396394	0.294381	0.044*	
O14	0.33696 (10)	0.45164 (5)	0.16404 (9)	0.0301 (2)	
H14A	0.303822	0.446857	0.101121	0.045*	
H14B	0.385680	0.424467	0.182002	0.045*	

### µ-Oxido-bis[(2-{[2-(3-methoxy-2-oxidobenzylideneamino)phenylimino]methyl}-6-methoxyphenolato)iron(III)] pentahydrate, (2) Atomic displacement parameters (Å^2^)

**Table:**

	*U*^11^	*U*^22^	*U*^33^	*U*^12^	*U*^13^	*U*^23^
C1	0.0127 (4)	0.0134 (5)	0.0128 (5)	0.0020 (4)	0.0055 (4)	0.0002 (4)
C2	0.0140 (5)	0.0171 (5)	0.0122 (5)	0.0019 (4)	0.0054 (4)	0.0004 (4)
C3	0.0170 (5)	0.0182 (5)	0.0163 (5)	0.0034 (4)	0.0076 (4)	0.0042 (4)
C4	0.0218 (6)	0.0149 (5)	0.0203 (6)	0.0039 (4)	0.0112 (5)	0.0034 (4)
C5	0.0227 (6)	0.0135 (5)	0.0180 (5)	0.0035 (4)	0.0108 (5)	0.0008 (4)
C6	0.0145 (5)	0.0142 (5)	0.0129 (5)	0.0009 (4)	0.0069 (4)	0.0002 (4)
C7	0.0170 (5)	0.0137 (5)	0.0137 (5)	−0.0001 (4)	0.0076 (4)	−0.0012 (4)
C8	0.0134 (4)	0.0145 (5)	0.0099 (4)	−0.0012 (4)	0.0055 (4)	−0.0012 (4)
C9	0.0180 (5)	0.0165 (5)	0.0134 (5)	−0.0001 (4)	0.0067 (4)	−0.0029 (4)
C10	0.0204 (6)	0.0214 (6)	0.0110 (5)	−0.0031 (4)	0.0059 (4)	−0.0036 (4)
C11	0.0194 (5)	0.0224 (6)	0.0105 (5)	−0.0018 (4)	0.0042 (4)	−0.0001 (4)
C12	0.0188 (5)	0.0175 (5)	0.0117 (5)	−0.0003 (4)	0.0045 (4)	0.0004 (4)
C13	0.0132 (5)	0.0146 (5)	0.0106 (4)	−0.0016 (4)	0.0053 (4)	−0.0016 (4)
C14	0.0124 (4)	0.0146 (5)	0.0125 (5)	0.0000 (4)	0.0054 (4)	0.0006 (4)
C15	0.0111 (4)	0.0125 (5)	0.0142 (5)	−0.0008 (4)	0.0060 (4)	−0.0013 (4)
C16	0.0138 (5)	0.0137 (5)	0.0182 (5)	0.0001 (4)	0.0076 (4)	0.0007 (4)
C17	0.0171 (5)	0.0133 (5)	0.0225 (6)	0.0003 (4)	0.0095 (5)	−0.0010 (4)
C18	0.0188 (5)	0.0156 (5)	0.0203 (6)	0.0000 (4)	0.0089 (5)	−0.0041 (4)
C19	0.0165 (5)	0.0153 (5)	0.0148 (5)	0.0000 (4)	0.0051 (4)	−0.0037 (4)
C20	0.0123 (4)	0.0122 (5)	0.0149 (5)	−0.0004 (4)	0.0055 (4)	−0.0019 (4)
C21	0.0178 (5)	0.0251 (6)	0.0114 (5)	0.0030 (5)	0.0040 (4)	0.0026 (4)
C22	0.0543 (11)	0.0246 (7)	0.0157 (6)	0.0062 (7)	0.0085 (7)	−0.0060 (5)
C23	0.0125 (4)	0.0116 (4)	0.0118 (4)	−0.0001 (4)	0.0055 (4)	−0.0001 (4)
C24	0.0153 (5)	0.0124 (5)	0.0114 (4)	−0.0004 (4)	0.0064 (4)	−0.0009 (4)
C25	0.0226 (6)	0.0138 (5)	0.0157 (5)	−0.0038 (4)	0.0082 (4)	−0.0026 (4)
C26	0.0280 (6)	0.0132 (5)	0.0179 (5)	−0.0043 (4)	0.0100 (5)	−0.0011 (4)
C27	0.0234 (6)	0.0136 (5)	0.0147 (5)	−0.0017 (4)	0.0088 (4)	0.0017 (4)
C28	0.0145 (5)	0.0132 (5)	0.0116 (4)	−0.0002 (4)	0.0058 (4)	0.0002 (4)
C29	0.0141 (5)	0.0143 (5)	0.0111 (4)	0.0013 (4)	0.0055 (4)	0.0026 (4)
C30	0.0124 (4)	0.0177 (5)	0.0095 (4)	0.0008 (4)	0.0049 (4)	0.0002 (4)
C31	0.0177 (5)	0.0229 (6)	0.0113 (5)	0.0005 (4)	0.0067 (4)	0.0024 (4)
C32	0.0237 (6)	0.0307 (7)	0.0092 (5)	−0.0010 (5)	0.0062 (4)	0.0017 (5)
C33	0.0274 (7)	0.0312 (7)	0.0106 (5)	−0.0046 (6)	0.0055 (5)	−0.0031 (5)
C34	0.0232 (6)	0.0222 (6)	0.0123 (5)	−0.0044 (5)	0.0054 (4)	−0.0033 (4)
C35	0.0140 (5)	0.0185 (5)	0.0105 (4)	0.0002 (4)	0.0053 (4)	−0.0003 (4)
C36	0.0131 (5)	0.0153 (5)	0.0135 (5)	−0.0014 (4)	0.0061 (4)	−0.0026 (4)
C37	0.0115 (4)	0.0125 (5)	0.0154 (5)	−0.0009 (4)	0.0057 (4)	−0.0017 (4)
C38	0.0146 (5)	0.0134 (5)	0.0212 (6)	−0.0012 (4)	0.0081 (4)	−0.0036 (4)
C39	0.0151 (5)	0.0123 (5)	0.0250 (6)	−0.0006 (4)	0.0085 (5)	−0.0011 (4)
C40	0.0152 (5)	0.0131 (5)	0.0216 (6)	0.0006 (4)	0.0075 (4)	0.0039 (4)
C41	0.0135 (5)	0.0127 (5)	0.0157 (5)	−0.0010 (4)	0.0062 (4)	0.0010 (4)
C42	0.0112 (4)	0.0111 (5)	0.0150 (5)	−0.0006 (3)	0.0057 (4)	0.0005 (4)
C43	0.0179 (5)	0.0214 (6)	0.0106 (5)	−0.0021 (4)	0.0042 (4)	−0.0030 (4)
C44	0.0274 (7)	0.0201 (6)	0.0177 (6)	−0.0007 (5)	0.0095 (5)	0.0065 (5)
Fe1	0.01062 (7)	0.01160 (7)	0.00918 (7)	0.00124 (5)	0.00416 (5)	−0.00053 (5)
Fe2	0.01054 (7)	0.01061 (7)	0.00868 (7)	−0.00008 (5)	0.00452 (5)	0.00020 (5)
N1	0.0128 (4)	0.0142 (4)	0.0109 (4)	0.0003 (3)	0.0056 (3)	−0.0008 (3)
N2	0.0119 (4)	0.0137 (4)	0.0099 (4)	−0.0004 (3)	0.0046 (3)	−0.0015 (3)
N3	0.0117 (4)	0.0147 (4)	0.0091 (4)	−0.0003 (3)	0.0045 (3)	−0.0001 (3)
N4	0.0130 (4)	0.0142 (4)	0.0108 (4)	−0.0005 (3)	0.0050 (3)	−0.0007 (3)
O1	0.0199 (4)	0.0220 (5)	0.0120 (4)	0.0066 (3)	0.0023 (3)	−0.0010 (3)
O2	0.0167 (4)	0.0185 (4)	0.0124 (4)	0.0071 (3)	0.0021 (3)	−0.0019 (3)
O3	0.0212 (4)	0.0133 (4)	0.0125 (4)	0.0016 (3)	0.0039 (3)	−0.0011 (3)
O4	0.0348 (6)	0.0193 (5)	0.0120 (4)	0.0060 (4)	0.0042 (4)	−0.0036 (3)
O5	0.0195 (4)	0.0157 (4)	0.0092 (3)	−0.0034 (3)	0.0037 (3)	−0.0003 (3)
O6	0.0165 (4)	0.0121 (4)	0.0108 (3)	−0.0029 (3)	0.0043 (3)	0.0008 (3)
O7	0.0183 (4)	0.0111 (4)	0.0130 (4)	0.0019 (3)	0.0077 (3)	0.0009 (3)
O8	0.0267 (5)	0.0136 (4)	0.0138 (4)	0.0006 (3)	0.0089 (3)	0.0034 (3)
O9	0.0125 (4)	0.0141 (4)	0.0141 (4)	0.0020 (3)	0.0067 (3)	0.0027 (3)
O10	0.0333 (6)	0.0250 (5)	0.0253 (5)	0.0070 (4)	0.0174 (5)	0.0052 (4)
O11	0.0225 (5)	0.0417 (7)	0.0297 (6)	−0.0074 (5)	0.0078 (5)	0.0043 (5)
O12	0.0428 (8)	0.0570 (9)	0.0246 (6)	−0.0005 (7)	0.0058 (6)	0.0004 (6)
O13	0.0514 (7)	0.0186 (5)	0.0158 (4)	−0.0107 (5)	0.0103 (5)	−0.0012 (4)
O14	0.0358 (6)	0.0269 (6)	0.0267 (5)	−0.0038 (5)	0.0116 (5)	−0.0049 (4)

### µ-Oxido-bis[(2-{[2-(3-methoxy-2-oxidobenzylideneamino)phenylimino]methyl}-6-methoxyphenolato)iron(III)] pentahydrate, (2) Geometric parameters (Å, º)

**Table:**

C1—C2	1.4235 (16)	C27—C28	1.4219 (17)
C1—C6	1.4187 (16)	C28—C29	1.4332 (16)
C1—O2	1.3080 (14)	C29—H29	0.9500
C2—C3	1.3810 (17)	C29—N3	1.3017 (16)
C2—O1	1.3690 (15)	C30—C31	1.4008 (17)
C3—H3	0.9500	C30—C35	1.4081 (18)
C3—C4	1.4035 (19)	C30—N3	1.4173 (15)
C4—H4	0.9500	C31—H31	0.9500
C4—C5	1.3756 (18)	C31—C32	1.3833 (18)
C5—H5	0.9500	C32—H32	0.9500
C5—C6	1.4147 (17)	C32—C33	1.396 (2)
C6—C7	1.4385 (17)	C33—H33	0.9500
C7—H7	0.9500	C33—C34	1.3878 (19)
C7—N1	1.2983 (16)	C34—H34	0.9500
C8—C9	1.3994 (16)	C34—C35	1.3991 (17)
C8—C13	1.4102 (17)	C35—N4	1.4175 (15)
C8—N1	1.4196 (15)	C36—H36	0.9500
C9—H9	0.9500	C36—C37	1.4339 (17)
C9—C10	1.3851 (17)	C36—N4	1.3061 (16)
C10—H10	0.9500	C37—C38	1.4221 (17)
C10—C11	1.3961 (19)	C37—C42	1.4144 (17)
C11—H11	0.9500	C38—H38	0.9500
C11—C12	1.3867 (18)	C38—C39	1.3694 (18)
C12—H12	0.9500	C39—H39	0.9500
C12—C13	1.3972 (16)	C39—C40	1.4090 (19)
C13—N2	1.4132 (15)	C40—H40	0.9500
C14—H14	0.9500	C40—C41	1.3798 (17)
C14—C15	1.4367 (16)	C41—C42	1.4285 (17)
C14—N2	1.3000 (16)	C41—O8	1.3671 (15)
C15—C16	1.4184 (17)	C42—O7	1.3075 (14)
C15—C20	1.4107 (17)	C43—H43A	0.9800
C16—H16	0.9500	C43—H43B	0.9800
C16—C17	1.3733 (18)	C43—H43C	0.9800
C17—H17	0.9500	C43—O5	1.4236 (14)
C17—C18	1.3996 (19)	C44—H44A	0.9800
C18—H18	0.9500	C44—H44B	0.9800
C18—C19	1.3817 (18)	C44—H44C	0.9800
C19—C20	1.4224 (17)	C44—O8	1.4288 (15)
C19—O4	1.3716 (16)	Fe1—N1	2.1035 (10)
C20—O3	1.3208 (15)	Fe1—N2	2.0893 (10)
C21—H21A	0.9800	Fe1—O2	1.9373 (9)
C21—H21B	0.9800	Fe1—O3	1.9262 (9)
C21—H21C	0.9800	Fe1—O9	1.7835 (9)
C21—O1	1.4248 (15)	Fe2—N3	2.1232 (10)
C22—H22A	0.9800	Fe2—N4	2.1036 (10)
C22—H22B	0.9800	Fe2—O6	1.9374 (8)
C22—H22C	0.9800	Fe2—O7	1.9246 (9)
C22—O4	1.4252 (17)	Fe2—O9	1.7893 (8)
C23—C24	1.4231 (16)	O10—H10A	0.8701
C23—C28	1.4187 (16)	O10—H10B	0.8712
C23—O6	1.3143 (14)	O11—H11A	0.8692
C24—C25	1.3801 (17)	O11—H11B	0.8713
C24—O5	1.3667 (14)	O12—H12A	0.8699
C25—H25	0.9500	O12—H12B	0.8693
C25—C26	1.4064 (18)	O13—H13A	0.8693
C26—H26	0.9500	O13—H13B	0.8689
C26—C27	1.3715 (18)	O14—H14A	0.8697
C27—H27	0.9500	O14—H14B	0.8713
			
C6—C1—C2	117.96 (11)	C35—C30—N3	114.64 (10)
O2—C1—C2	117.67 (10)	C30—C31—H31	120.2
O2—C1—C6	124.34 (10)	C32—C31—C30	119.66 (13)
C3—C2—C1	121.37 (11)	C32—C31—H31	120.2
O1—C2—C1	113.20 (10)	C31—C32—H32	119.8
O1—C2—C3	125.42 (11)	C31—C32—C33	120.44 (12)
C2—C3—H3	120.0	C33—C32—H32	119.8
C2—C3—C4	119.98 (11)	C32—C33—H33	119.7
C4—C3—H3	120.0	C34—C33—C32	120.60 (12)
C3—C4—H4	119.9	C34—C33—H33	119.7
C5—C4—C3	120.13 (12)	C33—C34—H34	120.2
C5—C4—H4	119.9	C33—C34—C35	119.52 (13)
C4—C5—H5	119.5	C35—C34—H34	120.2
C4—C5—C6	121.00 (12)	C30—C35—N4	115.56 (10)
C6—C5—H5	119.5	C34—C35—C30	119.85 (11)
C1—C6—C7	122.68 (11)	C34—C35—N4	124.57 (11)
C5—C6—C1	119.53 (11)	C37—C36—H36	117.5
C5—C6—C7	117.61 (11)	N4—C36—H36	117.5
C6—C7—H7	117.3	N4—C36—C37	125.07 (11)
N1—C7—C6	125.44 (11)	C38—C37—C36	117.59 (11)
N1—C7—H7	117.3	C42—C37—C36	122.47 (10)
C9—C8—C13	119.53 (10)	C42—C37—C38	119.90 (11)
C9—C8—N1	125.39 (11)	C37—C38—H38	119.6
C13—C8—N1	115.09 (10)	C39—C38—C37	120.82 (12)
C8—C9—H9	119.9	C39—C38—H38	119.6
C10—C9—C8	120.12 (12)	C38—C39—H39	120.0
C10—C9—H9	119.9	C38—C39—C40	120.04 (12)
C9—C10—H10	119.9	C40—C39—H39	120.0
C9—C10—C11	120.19 (12)	C39—C40—H40	119.9
C11—C10—H10	119.9	C41—C40—C39	120.23 (12)
C10—C11—H11	119.8	C41—C40—H40	119.9
C12—C11—C10	120.37 (11)	C40—C41—C42	121.20 (11)
C12—C11—H11	119.8	O8—C41—C40	125.45 (11)
C11—C12—H12	120.0	O8—C41—C42	113.35 (10)
C11—C12—C13	119.93 (12)	C37—C42—C41	117.75 (10)
C13—C12—H12	120.0	O7—C42—C37	124.38 (11)
C8—C13—N2	115.54 (10)	O7—C42—C41	117.87 (11)
C12—C13—C8	119.74 (11)	H43A—C43—H43B	109.5
C12—C13—N2	124.71 (11)	H43A—C43—H43C	109.5
C15—C14—H14	117.3	H43B—C43—H43C	109.5
N2—C14—H14	117.3	O5—C43—H43A	109.5
N2—C14—C15	125.35 (11)	O5—C43—H43B	109.5
C16—C15—C14	117.21 (11)	O5—C43—H43C	109.5
C20—C15—C14	122.99 (10)	H44A—C44—H44B	109.5
C20—C15—C16	119.78 (11)	H44A—C44—H44C	109.5
C15—C16—H16	119.8	H44B—C44—H44C	109.5
C17—C16—C15	120.34 (12)	O8—C44—H44A	109.5
C17—C16—H16	119.8	O8—C44—H44B	109.5
C16—C17—H17	119.7	O8—C44—H44C	109.5
C16—C17—C18	120.55 (12)	N2—Fe1—N1	77.43 (4)
C18—C17—H17	119.7	O2—Fe1—N1	87.35 (4)
C17—C18—H18	119.9	O2—Fe1—N2	145.27 (4)
C19—C18—C17	120.12 (12)	O3—Fe1—N1	145.37 (4)
C19—C18—H18	119.9	O3—Fe1—N2	87.87 (4)
C18—C19—C20	120.83 (12)	O3—Fe1—O2	87.38 (4)
O4—C19—C18	125.46 (11)	O9—Fe1—N1	102.80 (4)
O4—C19—C20	113.71 (11)	O9—Fe1—N2	103.56 (4)
C15—C20—C19	118.36 (11)	O9—Fe1—O2	110.23 (4)
O3—C20—C15	123.91 (11)	O9—Fe1—O3	111.13 (4)
O3—C20—C19	117.71 (11)	N4—Fe2—N3	76.28 (4)
H21A—C21—H21B	109.5	O6—Fe2—N3	86.45 (4)
H21A—C21—H21C	109.5	O6—Fe2—N4	140.16 (4)
H21B—C21—H21C	109.5	O7—Fe2—N3	152.65 (4)
O1—C21—H21A	109.5	O7—Fe2—N4	86.70 (4)
O1—C21—H21B	109.5	O7—Fe2—O6	93.50 (4)
O1—C21—H21C	109.5	O9—Fe2—N3	98.98 (4)
H22A—C22—H22B	109.5	O9—Fe2—N4	108.21 (4)
H22A—C22—H22C	109.5	O9—Fe2—O6	109.82 (4)
H22B—C22—H22C	109.5	O9—Fe2—O7	106.68 (4)
O4—C22—H22A	109.5	C7—N1—C8	121.48 (10)
O4—C22—H22B	109.5	C7—N1—Fe1	124.40 (8)
O4—C22—H22C	109.5	C8—N1—Fe1	113.22 (7)
C28—C23—C24	117.46 (10)	C13—N2—Fe1	113.26 (8)
O6—C23—C24	118.26 (10)	C14—N2—C13	120.76 (10)
O6—C23—C28	124.23 (10)	C14—N2—Fe1	125.96 (8)
C25—C24—C23	121.45 (11)	C29—N3—C30	121.35 (10)
O5—C24—C23	113.70 (10)	C29—N3—Fe2	125.02 (8)
O5—C24—C25	124.86 (11)	C30—N3—Fe2	112.90 (7)
C24—C25—H25	119.8	C35—N4—Fe2	113.05 (8)
C24—C25—C26	120.31 (11)	C36—N4—C35	119.45 (10)
C26—C25—H25	119.8	C36—N4—Fe2	127.21 (8)
C25—C26—H26	120.0	C2—O1—C21	117.22 (10)
C27—C26—C25	120.02 (12)	C1—O2—Fe1	130.60 (8)
C27—C26—H26	120.0	C20—O3—Fe1	130.40 (8)
C26—C27—H27	119.7	C19—O4—C22	116.84 (11)
C26—C27—C28	120.58 (11)	C24—O5—C43	117.86 (10)
C28—C27—H27	119.7	C23—O6—Fe2	130.35 (7)
C23—C28—C27	120.14 (11)	C42—O7—Fe2	133.51 (8)
C23—C28—C29	122.82 (11)	C41—O8—C44	117.82 (10)
C27—C28—C29	116.91 (10)	Fe1—O9—Fe2	137.97 (5)
C28—C29—H29	117.5	H10A—O10—H10B	104.3
N3—C29—C28	125.10 (11)	H11A—O11—H11B	104.5
N3—C29—H29	117.5	H12A—O12—H12B	104.6
C31—C30—C35	119.93 (11)	H13A—O13—H13B	104.6
C31—C30—N3	125.43 (11)	H14A—O14—H14B	104.4
			
C1—C2—C3—C4	−0.1 (2)	C28—C29—N3—Fe2	−12.57 (17)
C1—C2—O1—C21	178.19 (11)	C30—C31—C32—C33	−0.1 (2)
C1—C6—C7—N1	−1.6 (2)	C30—C35—N4—C36	154.03 (11)
C2—C1—C6—C5	2.23 (18)	C30—C35—N4—Fe2	−20.17 (13)
C2—C1—C6—C7	−172.67 (12)	C31—C30—C35—C34	0.74 (18)
C2—C1—O2—Fe1	−169.87 (9)	C31—C30—C35—N4	179.21 (11)
C2—C3—C4—C5	0.2 (2)	C31—C30—N3—C29	11.48 (18)
C3—C2—O1—C21	−2.89 (19)	C31—C30—N3—Fe2	−159.20 (10)
C3—C4—C5—C6	0.9 (2)	C31—C32—C33—C34	0.0 (2)
C4—C5—C6—C1	−2.1 (2)	C32—C33—C34—C35	0.5 (2)
C4—C5—C6—C7	173.03 (12)	C33—C34—C35—C30	−0.8 (2)
C5—C6—C7—N1	−176.54 (12)	C33—C34—C35—N4	−179.16 (13)
C6—C1—C2—C3	−1.17 (18)	C34—C35—N4—C36	−27.58 (18)
C6—C1—C2—O1	177.79 (11)	C34—C35—N4—Fe2	158.22 (11)
C6—C1—O2—Fe1	12.13 (19)	C35—C30—C31—C32	−0.26 (19)
C6—C7—N1—C8	174.88 (11)	C35—C30—N3—C29	−169.33 (11)
C6—C7—N1—Fe1	−16.72 (18)	C35—C30—N3—Fe2	19.99 (12)
C8—C9—C10—C11	−1.7 (2)	C36—C37—C38—C39	−178.65 (11)
C8—C13—N2—C14	162.34 (11)	C36—C37—C42—C41	−179.83 (11)
C8—C13—N2—Fe1	−19.29 (13)	C36—C37—C42—O7	0.22 (19)
C9—C8—C13—C12	3.85 (18)	C37—C36—N4—C35	−179.01 (11)
C9—C8—C13—N2	−176.60 (11)	C37—C36—N4—Fe2	−5.72 (18)
C9—C8—N1—C7	3.76 (18)	C37—C38—C39—C40	−0.56 (19)
C9—C8—N1—Fe1	−165.84 (10)	C37—C42—O7—Fe2	6.29 (18)
C9—C10—C11—C12	2.3 (2)	C38—C37—C42—C41	2.55 (17)
C10—C11—C12—C13	0.2 (2)	C38—C37—C42—O7	−177.41 (11)
C11—C12—C13—C8	−3.25 (19)	C38—C39—C40—C41	0.30 (19)
C11—C12—C13—N2	177.25 (12)	C39—C40—C41—C42	1.44 (18)
C12—C13—N2—C14	−18.13 (18)	C39—C40—C41—O8	−178.67 (12)
C12—C13—N2—Fe1	160.24 (10)	C40—C41—C42—C37	−2.84 (17)
C13—C8—C9—C10	−1.37 (18)	C40—C41—C42—O7	177.12 (11)
C13—C8—N1—C7	−175.78 (11)	C40—C41—O8—C44	−2.36 (18)
C13—C8—N1—Fe1	14.62 (13)	C41—C42—O7—Fe2	−173.67 (8)
C14—C15—C16—C17	−179.59 (11)	C42—C37—C38—C39	−0.91 (18)
C14—C15—C20—C19	179.96 (11)	C42—C41—O8—C44	177.53 (11)
C14—C15—C20—O3	1.53 (18)	N1—C8—C9—C10	179.10 (12)
C15—C14—N2—C13	−179.02 (11)	N1—C8—C13—C12	−176.57 (11)
C15—C14—N2—Fe1	2.83 (17)	N1—C8—C13—N2	2.98 (15)
C15—C16—C17—C18	0.26 (19)	N1—Fe1—O9—Fe2	−129.67 (7)
C15—C20—O3—Fe1	−19.60 (18)	N2—C14—C15—C16	−175.49 (11)
C16—C15—C20—C19	1.76 (17)	N2—C14—C15—C20	6.27 (19)
C16—C15—C20—O3	−176.67 (11)	N2—Fe1—O9—Fe2	−49.74 (8)
C16—C17—C18—C19	0.2 (2)	N3—C30—C31—C32	178.89 (12)
C17—C18—C19—C20	0.3 (2)	N3—C30—C35—C34	−178.50 (11)
C17—C18—C19—O4	179.85 (13)	N3—C30—C35—N4	−0.02 (15)
C18—C19—C20—C15	−1.27 (18)	N3—Fe2—O9—Fe1	−141.18 (7)
C18—C19—C20—O3	177.26 (12)	N4—C36—C37—C38	177.73 (12)
C18—C19—O4—C22	5.0 (2)	N4—C36—C37—C42	0.06 (19)
C19—C20—O3—Fe1	161.96 (9)	N4—Fe2—O9—Fe1	−62.77 (8)
C20—C15—C16—C17	−1.28 (18)	O1—C2—C3—C4	−178.89 (12)
C20—C19—O4—C22	−175.42 (13)	O2—C1—C2—C3	−179.31 (12)
C23—C24—C25—C26	0.5 (2)	O2—C1—C2—O1	−0.35 (17)
C23—C24—O5—C43	−173.78 (11)	O2—C1—C6—C5	−179.77 (12)
C23—C28—C29—N3	−5.11 (19)	O2—C1—C6—C7	5.33 (19)
C24—C23—C28—C27	1.92 (17)	O2—Fe1—O9—Fe2	138.43 (7)
C24—C23—C28—C29	−173.76 (11)	O3—Fe1—O9—Fe2	43.26 (8)
C24—C23—O6—Fe2	−164.34 (8)	O4—C19—C20—C15	179.11 (11)
C24—C25—C26—C27	1.1 (2)	O4—C19—C20—O3	−2.36 (17)
C25—C24—O5—C43	6.24 (18)	O5—C24—C25—C26	−179.53 (12)
C25—C26—C27—C28	−1.1 (2)	O6—C23—C24—C25	−179.54 (12)
C26—C27—C28—C23	−0.4 (2)	O6—C23—C24—O5	0.47 (16)
C26—C27—C28—C29	175.50 (13)	O6—C23—C28—C27	179.34 (12)
C27—C28—C29—N3	179.08 (12)	O6—C23—C28—C29	3.66 (19)
C28—C23—C24—C25	−1.96 (18)	O6—Fe2—O9—Fe1	129.37 (7)
C28—C23—C24—O5	178.06 (10)	O7—Fe2—O9—Fe1	29.25 (8)
C28—C23—O6—Fe2	18.26 (18)	O8—C41—C42—C37	177.26 (10)
C28—C29—N3—C30	177.92 (11)	O8—C41—C42—O7	−2.78 (16)

### µ-Oxido-bis[(2-{[2-(3-methoxy-2-oxidobenzylideneamino)phenylimino]methyl}-6-methoxyphenolato)iron(III)] pentahydrate, (2) Hydrogen-bond geometry (Å, º)

**Table:**

*D*—H···*A*	*D*—H	H···*A*	*D*···*A*	*D*—H···*A*
C7—H7···O10^i^	0.95	2.60	3.5233 (16)	165
C36—H36···O14^ii^	0.95	2.45	3.3930 (17)	172
C44—H44*B*···O11^iii^	0.98	2.58	3.4643 (19)	151
O10—H10*A*···O1	0.87	2.21	2.9342 (15)	140
O10—H10*A*···O2	0.87	2.28	3.0393 (14)	146
O10—H10*B*···O3	0.87	2.42	3.1432 (14)	141
O10—H10*B*···O4	0.87	2.28	3.0579 (15)	148
O11—H11*A*···O3	0.87	2.11	2.9635 (15)	169
O11—H11*B*···O1	0.87	2.48	3.2974 (16)	157
O11—H11*B*···O2	0.87	2.52	3.0582 (15)	121
O12—H12*A*···O10	0.87	2.00	2.8520 (19)	167
O12—H12*B*···O11	0.87	2.23	2.9457 (19)	139
O13—H13*A*···O5	0.87	2.11	2.8255 (14)	139
O13—H13*A*···O6	0.87	2.27	3.0237 (13)	145
O13—H13*B*···O7	0.87	2.30	3.0580 (14)	146
O13—H13*B*···O8	0.87	2.08	2.8108 (14)	141
O14—H14*A*···O12^iv^	0.87	1.99	2.8457 (17)	167
O14—H14*B*···O13	0.87	2.01	2.8683 (18)	167

Symmetry codes: (i) *x*, −*y*+1/2, *z*−1/2; (ii) −*x*+1, −*y*+1, −*z*+1; (iii) −*x*+2, −*y*+1, −*z*+1; (iv) *x*−1, *y*, *z*−1.
